# A prognostic model for *Schistosoma japonicum* infection-associated liver hepatocellular carcinoma: strengthening the connection through initial biological experiments

**DOI:** 10.1186/s13027-024-00569-4

**Published:** 2024-03-21

**Authors:** Shuyan Sheng, Bangjie Chen, Ruiyao Xu, Yanxun Han, Deshen Mao, Yuerong Chen, Conghan Li, Wenzhuo Su, Xinyang Hu, Qing Zhao, Scott Lowe, Yuting Huang, Wei Shao, Yong Yao

**Affiliations:** 1 First Clinical Medical College (First Affiliated Hospital), Anhui Medical University, Hefei, 230032 China; 2 Department of Microbiology and Parasitology, Anhui Provincial Laboratory of Pathogen Biology, School of Basic Medical Sciences, Anhui Medical University, Hefei, 230032 China; 3 Second Clinical Medical College, Anhui Medical University, Hefei, 230032 China; 4 College of Osteopathic Medicine, Kansas City University, 1750 Independence Ave, Kansas City, MO 64106 USA; 5 Division of Gastroenterology and Hepatology, Mayo Clinic in Florida, Jacksonville, FL USA; 6 School of Life Sciences, Anhui Medical University, Hefei, 230032 China

**Keywords:** *Schistosoma japonicum*, Liver hepatocellular carcinoma, Chronic liver disease, Prognostic signature

## Abstract

**Background:**

Numerous studies have shown that *Schistosoma japonicum* infection correlates with an increased risk of liver hepatocellular carcinoma (LIHC). However, data regarding the role of this infection in LIHC oncogenesis are scarce. This study aimed to investigate the potential mechanisms of hepatocarcinogenesis associated with *Schistosoma japonicum* infection.

**Methods:**

By examining chronic liver disease as a mediator, we identified the genes contributing to *Schistosoma japonicum* infection and LIHC. We selected 15 key differentially expressed genes (DEGs) using weighted gene co-expression network analysis (WGCNA) and random survival forest models. Consensus clustering revealed two subgroups with distinct prognoses. Least Absolute Shrinkage and Selection Operator (LASSO) and Cox regression identified six prognostic DEGs, forming an *Schistosoma japonicum* infection-associated signature for strong prognosis prediction. This signature, which is an independent LIHC risk factor, was significantly correlated with clinical variables. Four DEGs, including BMI1, were selected based on their protein expression levels in cancerous and normal tissues. We confirmed BMI1's role in LIHC using *Schistosoma japonicum*-infected mouse models and molecular experiments.

**Results:**

We identified a series of DEGs that mediate schistosomiasis, the parasitic disease caused by *Schistosoma japonicum* infection*,* and hepatocarcinogenesis, and constructed a suitable prognostic model. We analyzed the mechanisms by which these DEGs regulate disease and present the differences in prognosis between the different genotypes. Finally, we verified our findings using molecular biology experiments.

**Conclusion:**

Bioinformatics and molecular biology analyses confirmed a relationship between schistosomiasis and liver hepatocellular cancer. Furthermore, we validated the role of a potential oncoprotein factor that may be associated with infection and carcinogenesis. These findings enhance our understanding of *Schistosoma japonicum* infection's role in LIHC carcinogenesis.

**Supplementary Information:**

The online version contains supplementary material available at 10.1186/s13027-024-00569-4.

## Background

Liver cancer is the fifth most commonly diagnosed cancer and the most frequent cause of cancer-related deaths worldwide [3, 34]; it is the second most common cancer in China [10]. Despite the increasing incidence of liver cancer, the limited availability of treatment options remains a concern. In addition to physical methods, such as radiation, transplantation, and surgery, only a few approved medical therapy methods have been proposed, including a range of costly failures and a very small number of drug candidates [16]. Owing to the high heterogeneity of liver hepatocellular carcinoma (LIHC) [31], it is widely accepted that future medical treatments should focus on individualized care. However, implementing personalized care relies on our understanding of the risk factors that induce hepatocarcinogenesis and advanced detection of oncogenic alterations.

Liver cancer is typically caused by an underlying disease; however, the causes of these diseases vary significantly worldwide [16]. Among LIHC risk factors, large-scale transmission of schistosomiasis should not be overlooked, as it may have a lasting negative impact. Notably, schistosomiasis differs from bacterial and viral infections in that it is usually not an acute disease; rather, it is a chronic disease that gradually deteriorates health, leading to high morbidity and mortality [2]. These characteristics align with those of chronic diseases that induce primary liver cancer, and the ability of schistosomiasis to trigger LIHC has been widely recognized, both epidemiologically and pathologically. Epidemiological evidence from China and Japan supports *Schistosoma japonicum* infection, a major schistosomiasis-causing parasite, as an LIHC risk factor [23]. Kojiro et al. reported that autopsies revealed significantly higher rates of hepatocellular carcinoma in patients with chronic schistosomiasis than in healthy individuals [26]. In a series of studies, Inaba et al. suggested that a combination of schistosomiasis and other factors, such as hepatitis B virus infection and alcohol abuse, may contribute to LIHC. Filgueira et al. found that infection with *Schistosoma mansoni,* another schistosomiasis-causing parasite, can promote LIHC development as a single factor, even in the absence of other risk factors [15]. Although many aspects of LIHC development induced by *Schistosoma japonicum* infection remain unclear, several high-quality studies have been conducted. El-Tonsy et al. published a groundbreaking study confirming the role of schistosomiasis infection in promoting tumor growth [13]. By exploring schistosomiasis mechanisms at the tissue level, infection with *Schistosoma* was found to reduce the ability of the liver to process carcinogens [7, 22]. Roderfeld et al. [42] revealed that substances emanating from Schistosoma eggs, when ensnared within the liver tissue, trigger a lasting activation of proto-oncogenes linked to LIHC, such as C-jun, along with associated transcription factors, such as STAT3.

In recent years, bioinformatics has been considered an effective means to assess the relationship between specific factors and tumors [19, 20, 33]. In this study, we used the weighted gene co-expression network analysis (WGCNA) algorithm to systematically analyze the functions of differentially expressed genes (DEGs) caused by *Schistosoma japonicum* infection that are believed to be associated with LIHC. We screened the most representative DEGs using random survival forest models and the Least Absolute Shrinkage and Selection Operator (LASSO) regression algorithm and classified them using the non-negative matrix factorization (NMF) algorithm to assess differences in prognosis, immune cells, and tumor stromal scores across cluster subtypes. Subsequently, a prognostic signature of *Schistosoma japonicum* infection-associated LIHC was constructed and validated. The risk signature based on DEGs demonstrated a strong potential for survival prediction in *Schistosoma japonicum* infection-associated LIHC. Moreover, a nomogram integrating risk signature and clinical characteristics accurately predicted the prognosis of *Schistosoma japonicum* infection-associated LIHC patients. In addition, we assessed the potential response to immunotherapy and chemotherapy in distinct patient cohorts stratified based on the signature associated with *Schistosoma japonicum* infection. Furthermore, we created a mouse model of *Schistosoma japonicum* infection and examined the expression of relevant genes in liver samples using immunohistochemistry. A series of oncological experiments confirmed the important roles of DEGs in LIHC.

## Methods

### Criteria for selection and data acquisition

Gene expression data for both schistosomiasis-infected and healthy samples were obtained from the GEO-GSE61376 database (http://www.ncbi.nlm.nih.gov/geo). Furthermore, we acquired RNA sequencing data and the corresponding clinical datasets for individuals diagnosed with LIHC from TCGA (https://portal.gdc.cancer.gov/) and ICGC (https://dcc.icgc.org/). The detailed information was presented in Tables 1 and 2. Besides, Additional files 7, 8: supplementary table 1 and 2 were presented to describe the basic clinicopathological information from different databases.

**Table 1:** Statistical analysis and the Chi-square test of clinical-pathological information in high and low risk groups of TCGA

Covariates	Type	Total	High	Low	*p* Value
Grade	G1	55 (14.86%)	20 (10.81%)	35 (18.92%)	0
Grade	G2	177 (47.84%)	70 (37.84%)	107 (57.84%)	
Grade	G3	121 (32.7%)	83 (44.86%)	38 (20.54%)	
Grade	G4	12 (3.24%)	10 (5.41%)	2 (1.08%)	
Grade	Unknow	5 (1.35%)	2 (1.08%)	3 (1.62%)	
Stage	Stage I	171 (46.22%)	75 (40.54%)	96 (51.89%)	0.0332
Stage	Stage II	85 (22.97%)	47 (25.41%)	38 (20.54%)	
Stage	Stage III	85 (22.97%)	51 (27.57%)	34 (18.38%)	
Stage	Stage IV	5 (1.35%)	1 (0.54%)	4 (2.16%)	
Stage	Unknow	24 (6.49%)	11 (5.95%)	13 (7.03%)	
M	M0	266 (71.89%)	137 (74.05%)	129 (69.73%)	0.5832
M	M1	4 (1.08%)	1 (0.54%)	3 (1.62%)	
M	Unknow	100 (27.03%)	47 (25.41%)	53 (28.65%)	
N	N0	252 (68.11%)	130 (70.27%)	122 (65.95%)	0.6704
N	N1	4 (1.08%)	3 (1.62%)	1 (0.54%)	
N	Unknow	114 (30.81%)	52 (28.11%)	62 (33.51%)	
T	T1	181 (48.92%)	79 (42.7%)	102 (55.14%)	0.0591
T	T2	93 (25.14%)	53 (28.65%)	40 (21.62%)	
T	T3	80 (21.62%)	44 (23.78%)	36 (19.46%)	
T	T4	13 (3.51%)	9 (4.86%)	4 (2.16%)	
T	Unknow	3 (0.81%)	0 (0%)	3 (1.62%)	
Age	< = 61	192 (51.89%)	96 (51.89%)	96 (51.89%)	1
Age	> 61	178 (48.11%)	89 (48.11%)	89 (48.11%)	
Gender	Female	121 (32.7%)	58 (31.35%)	63 (34.05%)	0.6576
Gender	Male	249 (67.3%)	127 (68.65%)	122 (65.95%)

**Table 2:** Statistical analysis and the Chi-square test of clinical-pathological information in high and low risk groups of ICGC

Covariates	Type	Total	High	Low	*P* value
Gender	Female	61 (26.41%)	36 (31.3%)	25 (21.55%)	0.1255
Gender	Male	170 (73.59%)	79 (68.7%)	91 (78.45%)	
Stage	1	36 (15.58%)	14 (12.17%)	22 (18.97%)	0.3173
Stage	2	105 (45.45%)	51 (44.35%)	54 (46.55%)	
Stage	3	71 (30.74%)	38 (33.04%)	33 (28.45%)	
Stage	4	19 (8.23%)	12 (10.43%)	7 (6.03%)	
Age	< = 69	124 (53.68%)	60 (52.17%)	64 (55.17%)	0.7452
Age	> 69	107 (46.32%)	55 (47.83%)	52 (44.83%)	

### Construction of a gene co-expression network

We used the R-based WGCNA package (https://horvath.genetics.ucla.edu/html/CoexpressionNetwork/Rpackages/WGCNA/) to construct the gene coexpression network. We first selected the top 25% of genes with the highest variance from GSE61376 for network construction. Using the pickSoftThreshold function and dynamic tree-cutting algorithm of the WGCNA package, we calculated *p*-values and partitioned different modules. The WGCNA optimal soft threshold was calculated using the pickSoftThreshold function. The soft threshold with a signed R^2^ > 0.9 (first time) was selected as the optimal soft threshold. The result was stored in the powerEstimate slot of the pickSoftThreshold function and was automatically chosen. The correlation between module feature genes and clinical features was analyzed using Pearson's correlation coefficient. The correlation between gene expression and clinical information was assessed using the gene significance (GS). Key modules were identified by recognizing the GS of all the genes within each module.

### Random survival forest models

Data related to LIHC from TCGA were extracted for constructing random survival forest models using the rfsrc function in the “random forest SRC” package. The random survival forest technique trains a considerable number of surviving trees and combines predictions from these individual trees using a voting mechanism to produce the final results. By default, the model created 1000 binary survival trees. As the number of surviving trees continued to increase, the error rate curve stabilized at a certain point, suggesting that the chosen number of trees was suitable.

### Identifying molecular subtypes using NMF

Tumor molecular classification, proposed by the National Cancer Institute, has shifted from morphology-based to molecular-based tumor classification. This approach recognizes tumors as a class of disease, acknowledging their high heterogeneity in histopathology and molecular biology. It utilizes gene clusters to describe tumor characteristics because of the complex nature of tumors involving multiple genes. This shift aids in precise diagnosis, prognosis stratification, treatment guidance, and drug development. Technological advancements in molecular biology support this transition and facilitate the development of individualized targeted therapies. NMF clustering was used to group LIHC samples. To select various numerical values for the NMF, we referred to previous studies [32, 51]. The NMF algorithm is typically used to determine the number of categories. Its basic principle is to decompose the data matrix into the product of two non-negative matrices, where one matrix represents the importance weights of features and the other represents the expression of samples on these features. The number of categories that capture the data structure most effectively can be determined by adjusting the dimensions of the decomposed matrices. We first used different numbers of categories for NMF decomposition and selected the optimal number of categories by comparing the goodness-of-fit indicators of the models. Furthermore, to facilitate the observation of the optimal classification, we visualized the decomposed matrices using heat maps. The criterion for determining the optimal rank value was the first point at which the maximum change in the cophenetic value occurred as K varied. In the above results, the cophenetic value showed the greatest change at ranks 2–3, and so the optimal rank value was chosen as 2.

### Construction and validation of the prognostic signature

We used a cohort from TCGA as the training set and an ICGC cohort for external validation. Univariate Cox proportional hazard regression analysis and LASSO filtration were used to identify prognosis-related genes (*p* < 0.05), implemented via the "glmnet" package. The glmnet package adapts to generalized linear and similar models through penalized maximum likelihood. The parameter controlling the LASSO regression or elastic net regression on a logarithmic scale is the regularization parameter lambda. When mentioning the LASSO regression, one cannot ignore the ridge regression and Elastic Net. An alpha of 0 represents ridge regression without variable selection. Alpha = 1 represents a LASSO regression with variable selection. Alpha values between [0,1] represent Elastic Net regression. Therefore, the value of the alpha parameter was set to 1. The multivariate Cox proportional hazards model regarded six genes (FAM98A, UBE2E1, NOP56, GHR, TMEM106C, and BMI1) as immune-related risk signatures, defined as follows: risk score = (level of gene a × coefficient a) + (level of gene b × coefficient b) + (level of gene c × coefficient c) + … + (level of gene n × coefficient n). In our model, the risk score reflected the prognosis of patients with LIHC, with lower scores indicating a better prognosis. Patients were stratified into high- and low-risk groups using the median risk score as the cut-off value. We evaluated the predictive performance using Kaplan–Meier survival curves, and statistical significance was determined using log-rank p-values, employing the “survival” and “survminer” packages. For additional assessments, we employed time-dependent receiver operating characteristic (ROC) curves to measure the predictive capability of the signature using the survivalROC package.

### Independence validation of the prognostic model

We used both univariate and multivariate Cox regression analyses to investigate whether the risk score was an independent prognostic factor for LIHC in each independent cohort (TCGA and ICGC).

### Construction and evaluation of the nomogram

A nomogram was established using R package "repglot” and assessed by time-dependent ROC curves with the R package "pROC,” the Concordance index (C-index), and decision curve analysis (DCA). The data were obtained from the ICGC-LIRI-JP.

### Expression difference and copy number variation (CNV) frequency analysis

To investigate the differential expression of FAM98A, UBE2E1, NOP56, GHR, TMEM106C, and BMI1 between normal liver and LIHC tissues, we utilized publicly available transcriptomic datasets from TCGA. Raw RNA sequencing data were downloaded and pre-processed using the R package, TCGAbiolinks. Data normalization and gene-level quantification were performed using the DESeq2 package. Differential gene expression analysis between the normal liver and LIHC tissues was conducted using the edgeR package.

To investigate the CNV frequency of these genes, we used the cBioPortal database to analyze the CNV data for FAM98A, UBE2E1, NOP56, GHR, TMEM106C, and BMI1 in LIHC patient samples. CNV data, including the number of samples with gene amplification, gene deletion, and normal copy number status, were obtained from cBioPortal. We calculated the CNV frequency for each gene, represented as the proportion of samples with gene amplifications or deletions out of the total number of samples.

### Immune cell infiltration and tumor microenvironment

The monotonic relationship between the risk scores and immune cell infiltration was explored using Spearman’s correlation analysis. Immune cell infiltration levels were determined using the TIMER, CIBERSORT, XCELL, QUANTISEQ, MCPcounter, EPIC, and CIBERSORT algorithms. High- and low-risk groups were analyzed using the Wilcoxon signed-rank test to compare immune cell composition. In addition, we computed tumor microenvironment (TME) scores for the entire LIHC cohort using the “ESTIMATE” package. Finally, we conducted a Spearman correlation test by intersecting transcription gene expression data with stemness scores (based on RNA expression) to further explore potential correlations.

### Evaluation of chemotherapy and immunotherapy sensitivity

We performed differential analysis of chemotherapy sensitivity using the Genomics of Drug Sensitivity database (https://www.cancerrxgene.org/). Additionally, we predicted the potential responses to immune checkpoint blockade (ICB) using a Tumor Immune Dysfunction and Exclusion (TIDE) algorithm.

### Functional enrichment analysis

We conducted gene set enrichment analysis (GSEA) of DEGs. To understand the functional categories (ontologies) associated with these DEGs, we utilized Gene Ontology (GO) and Kyoto Encyclopedia of Genes and Genomes (KEGG) pathway enrichment analysis.

### Immunohistochemical (IHC) staining from HPA

The protein levels of BMI1, FAM98A, NOP56, and UBE2E1 in normal liver and LIHC tissues were analyzed using the Tissue Atlas and Pathology Atlas sections of HPA (http://www.proteinatlas.org).

### Parasite infection

We sourced the *Schistosoma japonicum* cercariae from infected *Oncomelania hupensis* (snails) provided by the Jiangsu Center for Disease Control and Prevention in China. Wild-type (WT) C57BL/6 mice were infected via percutaneous exposure to an average of 40 ± 5 cercariae; uninfected WT mice served as the control group. To assess the outcomes of *Schistosoma japonicum* infection, we sacrificed all mice 10 weeks after the initial infection. All experimental protocols were approved by the Institutional Animal Care and Use Committee of Anhui Medical University.

### Cell lines and cell culture

Human normal liver cell line (MIHA) and LIHC cell lines (HepG2, Hep3B, and Huh-7 cells) were acquired from the Center for Excellence in Molecular Cell Science, Shanghai, China. All cell lines were initially preserved in liquid nitrogen and subsequently cultured in a humidified incubator at 37 °C with 5% CO_2_. The culture medium was Dulbecco's Modified Eagle's Medium (DMEM; Gibco BRL, USA), supplemented with 1% antibiotics (100 U/ml penicillin and 100 µg/ml streptomycin sulfates, sourced from Sigma, USA), and 10% heat-inactivated fetal bovine serum (FBS; Gibco, USA).

### RNA interference

We designed and synthesized BMI1-siRNA using GenePharma Corporation (Shanghai, China). The sequences for the BMI1-siRNA were as follows: 5’-CCGUCUUAAUUUUCCAUUG-3. Human LIHC cells (HepG2, Hep3B, and Huh-7 cells) in the logarithmic growth phase were seeded into 6-well plates, with each well containing a cell density of 6 × 10^5^ cells in antibiotic-free DMEM as the culture medium. Transfection was carried out using a Lipofectamine™2000 kit. Subsequently, the cells were incubated in a humidified incubator at 37°C with 5% CO_2_ for 24 h before being harvested for further analysis.

### IHC staining

The dewaxed sections were placed in Xylene I (60 min), 100% alcohol I (5 min), 100% alcohol II (5 min) 90% alcohol (5 min), 80% alcohol (5 min), 70% alcohol (5 min), and then pure water (3 min). The slides were then incubated with 3% hydrogen peroxide (H_2_O_2_) for 10 min. The antigen retrieval process was then carried out by incubating mouse brain tissue slides in citrate buffer (11.48 g citric acid, 16.75 g trisodium citrate and 100 ml ddH2O, OH = 6.0) for 30 min (heated in a microwave for 8 min, cooled for 2 min, boiled for 2 min, repaired for 5 min, and cooled naturally to room temperature). The slides were treated with rabbit polyclonal antibodies against BMI1, FAM98A, UBE2E1, and NOP56 (diluted 1:100, Abcam, UK) overnight after pre-incubation with 0.3% H_2_O_2_ and blocked with 5% goat serum. We used 3,3'-diaminobenzidine tetrahydrochloride (DBA) staining to visualize the results. The slides were then re-stained with hematoxylin for 5 min. The immune complexes were observed under a microscope after cleaning, drying, becoming transparent, and fixing using a gel. Three replicates were performed for each experiment.

### Flow cytometry assay

Cells were fixed in phosphate-buffered saline (PBS) with 75% ice-cold ethanol. Afterwards, the fixed cells were treated with bovine pancreatic RNase (2 mg/ml, Sigma) and propidium iodide (10 mg/ml, Invitrogen), and incubated for 30 min at room temperature while being protected from light. In the graphical representation, the four quadrants represent necrotic, viable, early stage apoptotic, and late-stage apoptotic cells. Cell cycle progression and apoptosis were detected using a flow cytometer (BD Biosciences, NJ, USA).

### Transwell assay

Hep3B and HepG2 cells were placed in the top chamber of the well in a serum-free medium at a density of 2 × 10^6^ cells per well. The lower chamber was filled with 500 l of culture medium containing 20% FBS. After incubation for 2 days in a 5% (v/v) CO_2_ incubator at room temperature, the cells and Matrigel in the top chamber that did not invade were removed. Cell fixation and other techniques were performed as described in our previous study [9].

### Wound healing assay

Cells were seeded at a concentration of 1 × 10^6^ cells/mm in six-well plates and incubated for 24 h, after which they reached approximately 70% confluency. To create a horizontal scratch, a gentle motion was applied to the bottom surface of the adherent cells using a 10 µL autoclaved pipette tip. The cells were carefully washed twice with PBS to remove detached cells. The plates were incubated for 24 h. Finally, the cells were fixed using a methanol solution and stained with crystal violet. We used an inverted microscope to observe scratch healing, and captured and documented images.

### CCK8 assay

Cells were seeded in 96-well microplates (Corning, Corning, NY, USA) at a concentration of 5 × 10^3^ cells per well in 100 L of culture medium. Cells were exposed to different concentrations of Tan-I (0, 1.2, 2.4, 4.8, and 9.6 g/mL). After 24 h, 10 L of the CCK-8 reagent was added to each well and left for an additional 2 h. The experiments were performed in triplicate, and the absorbance was measured at 450 nm using a microplate reader (Bio-Rad, Hercules, CA, USA). Wells without cells were used as blanks. Cell proliferation was quantified based on the obtained absorbance values.

### Statistical analysis

Between-group data were analyzed by one-way analysis of variance (ANOVA) using the SPSS software (version 20.0); p < 0.05 was considered statistically significant. GraphPad Prism (version 9.0) was used for image production, and data are presented as the mean ± S.D. All other statistical analyses were performed using R version 4.0.4 (Institute for Statistics and Mathematics, Vienna, Austria; https://www.r-project.org). Repeat values were averaged, and missing values were removed. The RNA-seq data were merged and normalized by using the “limma” package. Correlations were determined using Spearman correlation analysis. The Wilcoxon test and t-test were used to compare clinical variables. Survival status was assessed using Cox regression analysis. The ‘survival” and “survminer” packages was employed to perform Kaplan–Meier analyses. Overall survival (OS) was calculated using the Kaplan–Meier method and evaluated using the log-rank test. Two-tailed p < 0.05 was considered statistically significant. The sensitivity and specificity of the model were evaluated using ROC curves employing the “survivalROC” package. The heatmaps was created using the “pheatmap” package. In addition, we verified the confidence of the model using test datasets and entire datasets. Hazard ratios (HRs) and 95% confidence intervals (CIs) were used to describe the relative risk.

## Results

### Identification of DEGs

**Fig. 1:**
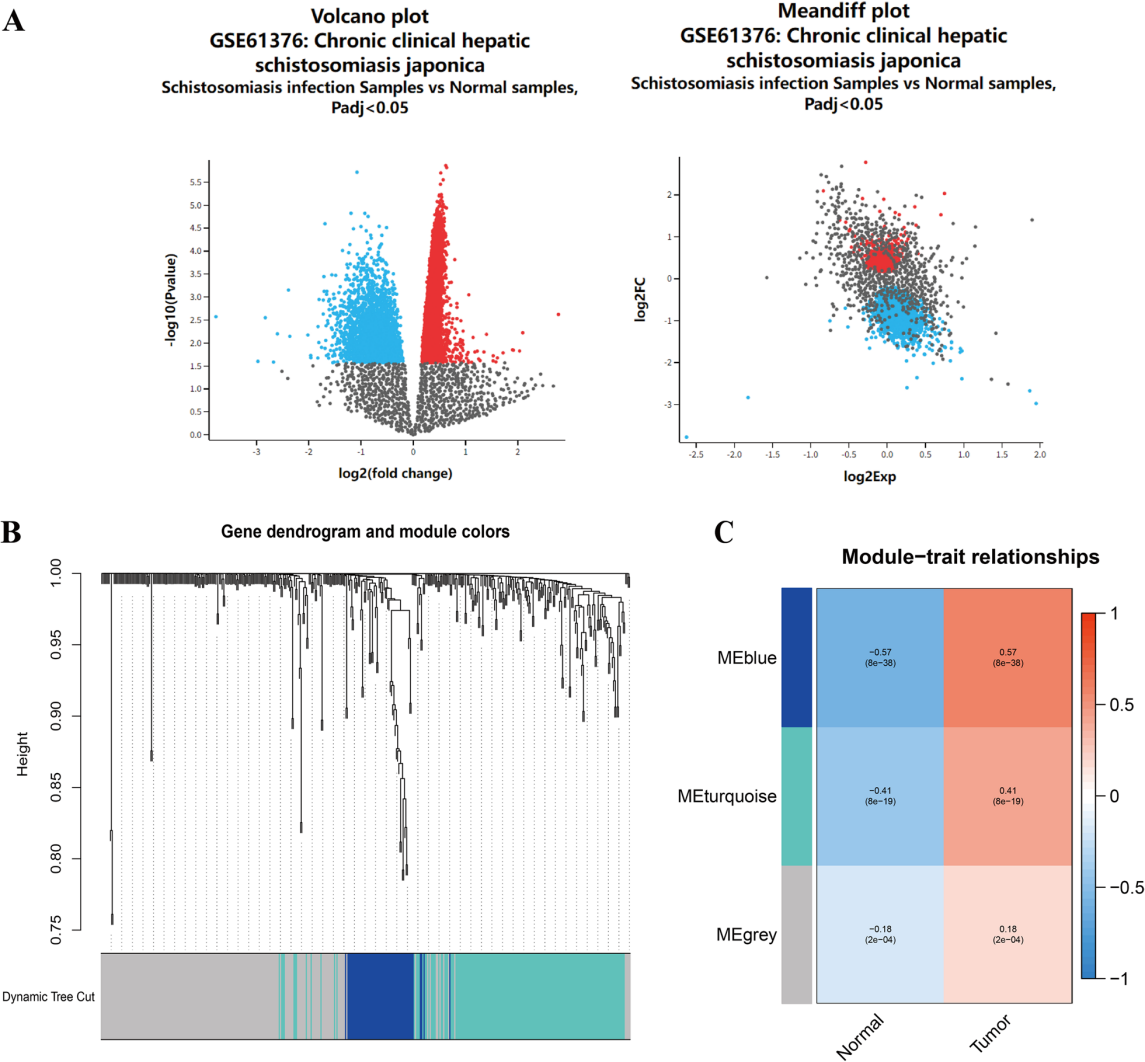
DEGs in schistosomiasis-induced cirrhotic liver tissue vs. normal liver tissue and liver cancer-associated schistosomiasis-related genes. **A** Volcano plot and Meandiff plot showing the DEGs between schistosomiasis infection samples and normal samples. **B** Cluster dendrogram of co-expression DEGs. **C** Heatmap illustrating correlations between modules and traits. Red indicates a positive correlation; blue indicates a negative correlation

In the GSE61376 dataset, 29,223 genes were identified. Among these, 454 DEGs were filtered using the criteria of p < 0.05 and |logFC|≥ 1 when comparing schistosomiasis infection samples to normal samples. This set of DEGs comprised of 432 upregulated and 22 downregulated genes. DEGs were visualized using a volcano plot and a mean difference plot (Fig. 1A).

### Construction of co-expression modules by WGCNA

Constructing a WGCNA of schistosomiasis infection and chronic liver disease is a powerful approach that integrates genomic data to discover potential oncogenic molecules that may be hidden among numerous genes. Using this approach, we constructed a co-expression network and identified modules within a dataset comprising 424 samples. To construct the network, we utilized the top 25% of the gene expression data and selected a power value of 3. The genes altered in schistosomiasis-induced chronic liver disease were divided into three modules. In the grey module, no co-expression patterns were observed. The blue and turquoise modules displayed a positive correlation with tumor development. Therefore, exploration of the genes in these two modules and their prognosis in liver cancer was performed using a random forest model (Fig. 1C).

### Random forest survival analysis

**Fig. 2:**
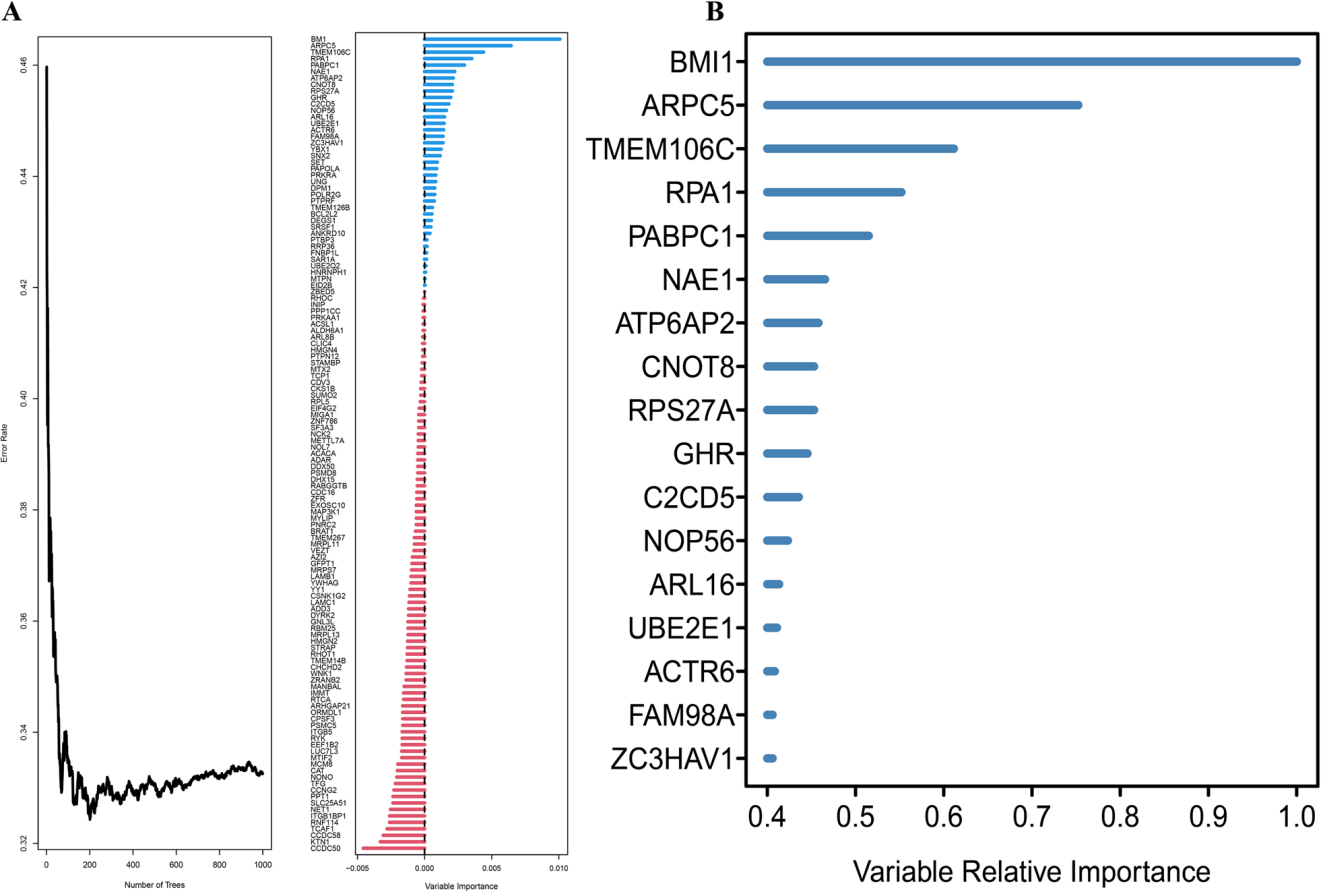
Schistosomiasis-related genes associated with prognosis by random survival forest models. **A** Error rate and variable importance of schistosomiasis-related genes. **B** Variable relative importance for predictors

To further select DEGs with prognostic value from the genes positively correlated with tumor development, a random forest-based approach was implemented for survival analysis. Figure 2A shows the association between the error rate and number of classification trees. We embraced and adopted the concept of WGCNA. Biological connections conform to this scale-free network distribution, known as the power-law distribution. In an organism, for a cell, many proteins or RNAs are expressed and have different functions. Some play extremely important roles, whereas others have minimal effects. The advantage of this distribution is that, even if some nodes are removed, the network can still function normally. Therefore, we used a relatively strict cutoff to filter out less important features, avoiding redundant features from affecting the final modeling. Ultimately, we decided to adopt 0.4 as the relative importance cutoff, and 17 genes were identified (Fig. 2B). These 17 DEGs not only indicate the occurrence of liver cancer but also have the potential to predict and evaluate the prognosis of liver cancer.

### Identification of disease subtypes using NMF

Using the NMF algorithm, we decomposed molecular subtypes based on the 17 genes. We identified two new liver cancer subtypes (Additional file 1: Fig. S1A). Patients with these subtypes exhibited significant differences in survival rates, including OS and disease-free interval (DFI). We investigated the reasons for these differences in patient survival from a microenvironmental perspective. The results indicate that patients with subtype C2 not only have higher immune cell content but also have an advantage in endothelial cell content. This suggests that the better prognosis of liver cancer patients with subtype C2 may be related to better antitumor immune function and endothelial homeostasis (Additional file 1: Fig. S1B and C).

### Immune and prognostic analysis of cluster subtypes

**Fig. 3:**
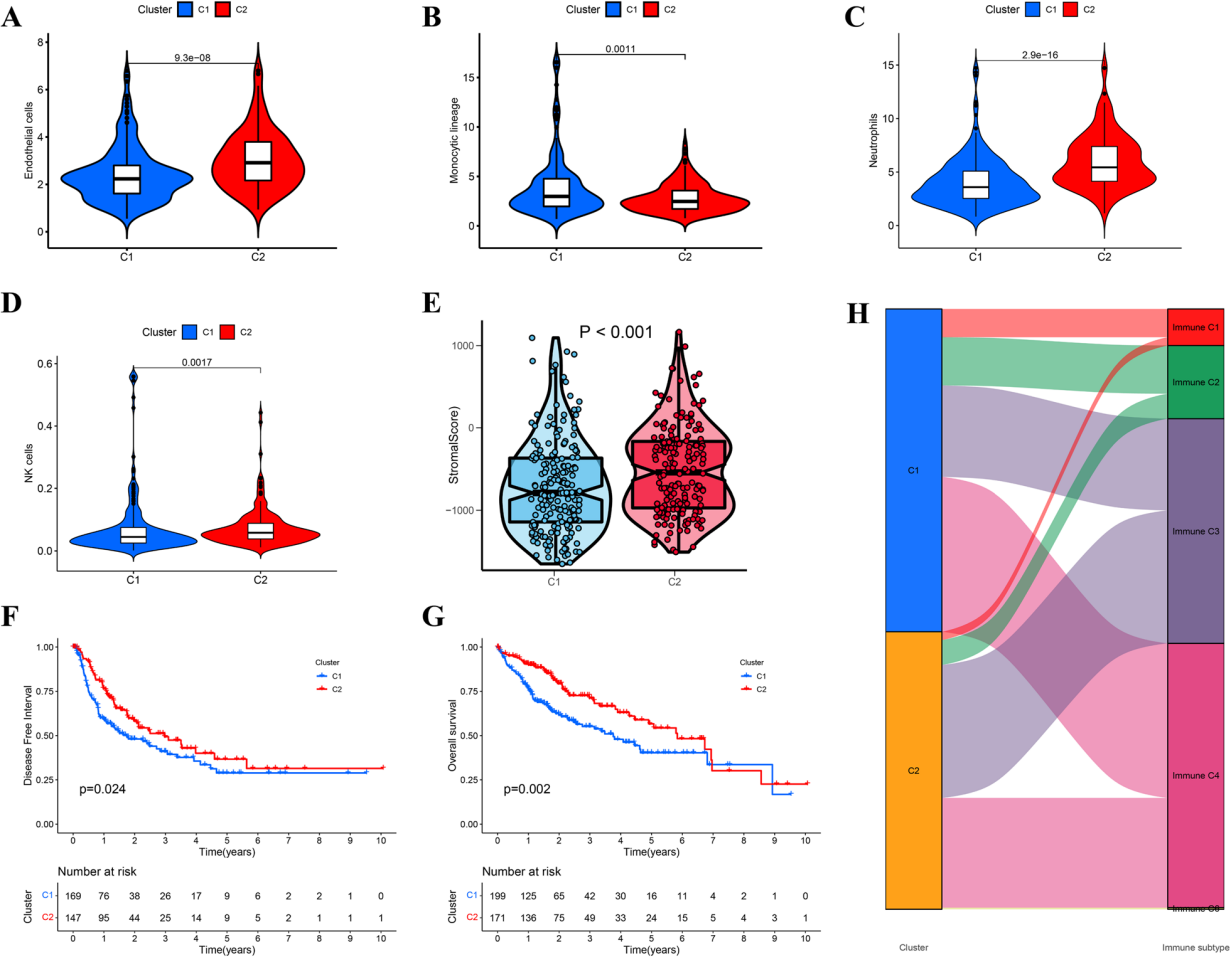
Differentiation analysis of clusters. **A**–**E** Differences of the endothelial cells, monocytic lineage, neutrophils, NK cells, and stromal scores in two clusters. **F** Disease free interval in the two clusters. **G** Overall survival in the two clusters. **H** Sankey diagram demonstrating distribution of immune subtypes between the two clusters

To assess the composition of immune cells in the distinct disease subgroups, we used the Wilcoxon test to compare the distribution of immune cells within these subgroups. Our analysis revealed that the monocytic lineage was more abundant in cluster 1, whereas endothelial cells, neutrophils, and natural killer (NK) cells were more prevalent in cluster 2 (Fig. 3A–D). Furthermore, the stromal score of cluster 1 was significantly lower than that of cluster 2 (Fig. 3E). Moreover, patients in cluster 2 exhibited a longer DFI (*p* = 0.024; Fig. 3F) and OS (*p* = 0.002; Fig. 3Gg) than patients in cluster 1.Finally, as shown in Fig. 3H, cluster 1 had a higher prevalence of the immune C1, C2, and C4 subtypes, whereas cluster 2 contained the immune C3 subtype.

### Prognostic signature construction

**Fig. 4:**
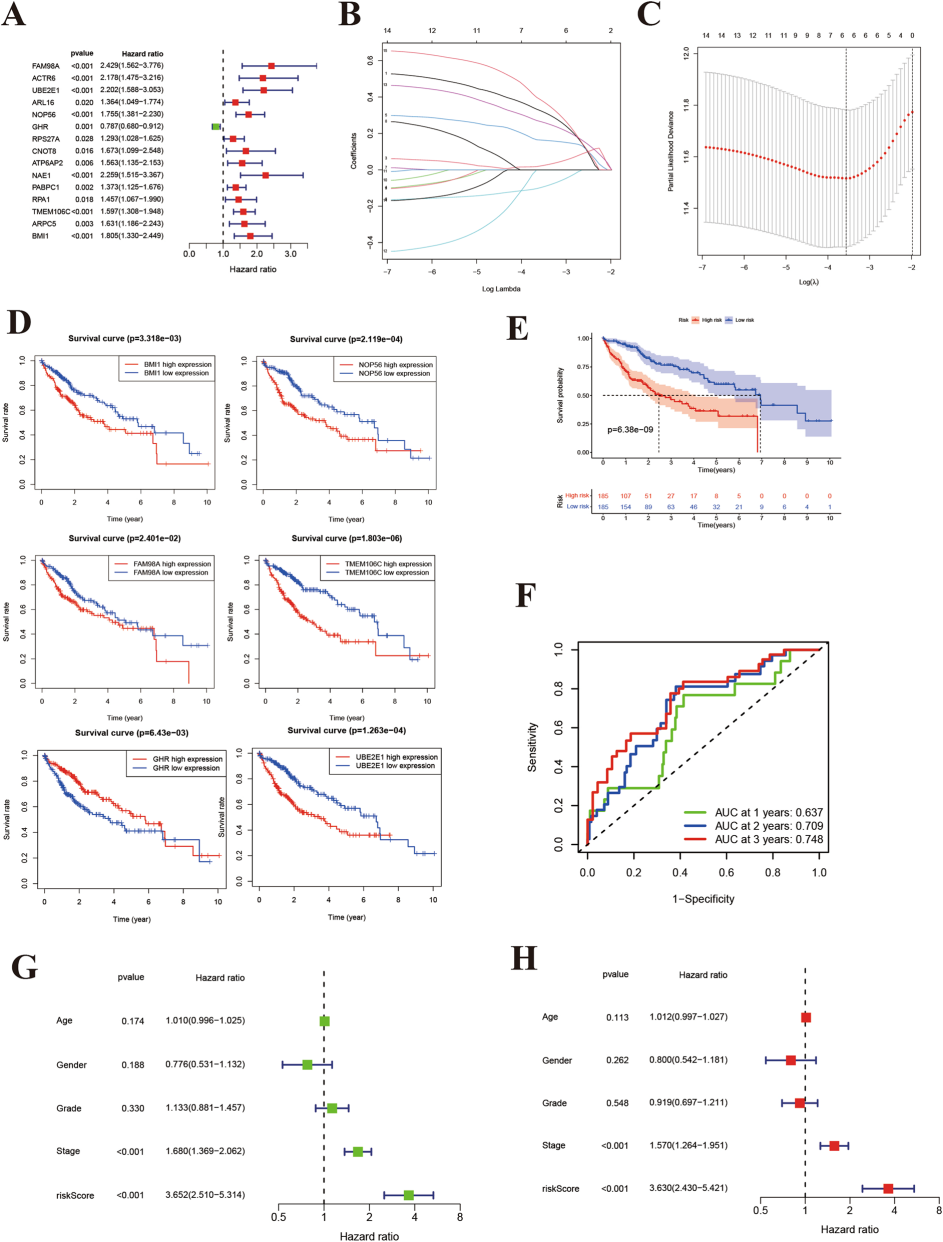
Constructing the LASSO–Cox model. **A** Forrest plot of prognostic genes. **B**, **C** LASSO-penalized Cox analysis conducted to build a prognostic model. **D** Kaplan–Meier survival analysis for six-gene signature, including FAM98A, NOP56, BMI1, GHR, TMEM106C, and UBE2E1. **E**, **F** Overall survival of patients in TCGA cohort. **G**, **H** Univariate and multivariate regression analyses showing the association between risk score and clinicopathological characteristics in relation to overall survival in TCGA cohort. Green represents univariate analysis results; red represents multivariate analysis results

Univariate Cox regression analysis identified 15 genes with a clear relationship with OS (Fig. 4A), including 14 prognostic risk factors (hazard ratio [HR] > 1) and one prognostic protective factor (HR < 1). Following LASSO regression analysis, six genes were incorporated into our prognostic signature (Fig. 4B, C): risk score = (0.38376 × BMI1) + (0.32374 × FAM98A) + (0.26462 × TMEM106C) + (0.16348 × NOP56) + (0.02153 × UBE2E1) + (− 0.09732 × GHR). We examined the relationship between the survival rate and expression levels of these six genes. Our analysis revealed that the high expression groups of FAM98A, UBE2E1, NOP56, TMEM106C, and BMI1 had lower survival rates than the low expression groups. In contrast, the high expression group of the GHR gene exhibited a higher survival rate, as shown in Fig. 4D. As shown in Fig. 4E, the high-risk group in the training set of TCGA had notably lower OS than the low-risk group (*p* = 6.38e−09). The AUC for predicting OS at 1, 2, and 3-years were 0.805, 0.709, and 0.709, respectively (Fig. 4F). Moreover, both univariate and multivariate Cox regression analyses demonstrated the independent predictive ability of the risk score (Fig. 4G, H). These results provide initial evidence supporting the usefulness of classifying patient prognosis using risk scores.

### Prognostic signature validation

**Fig. 5:**
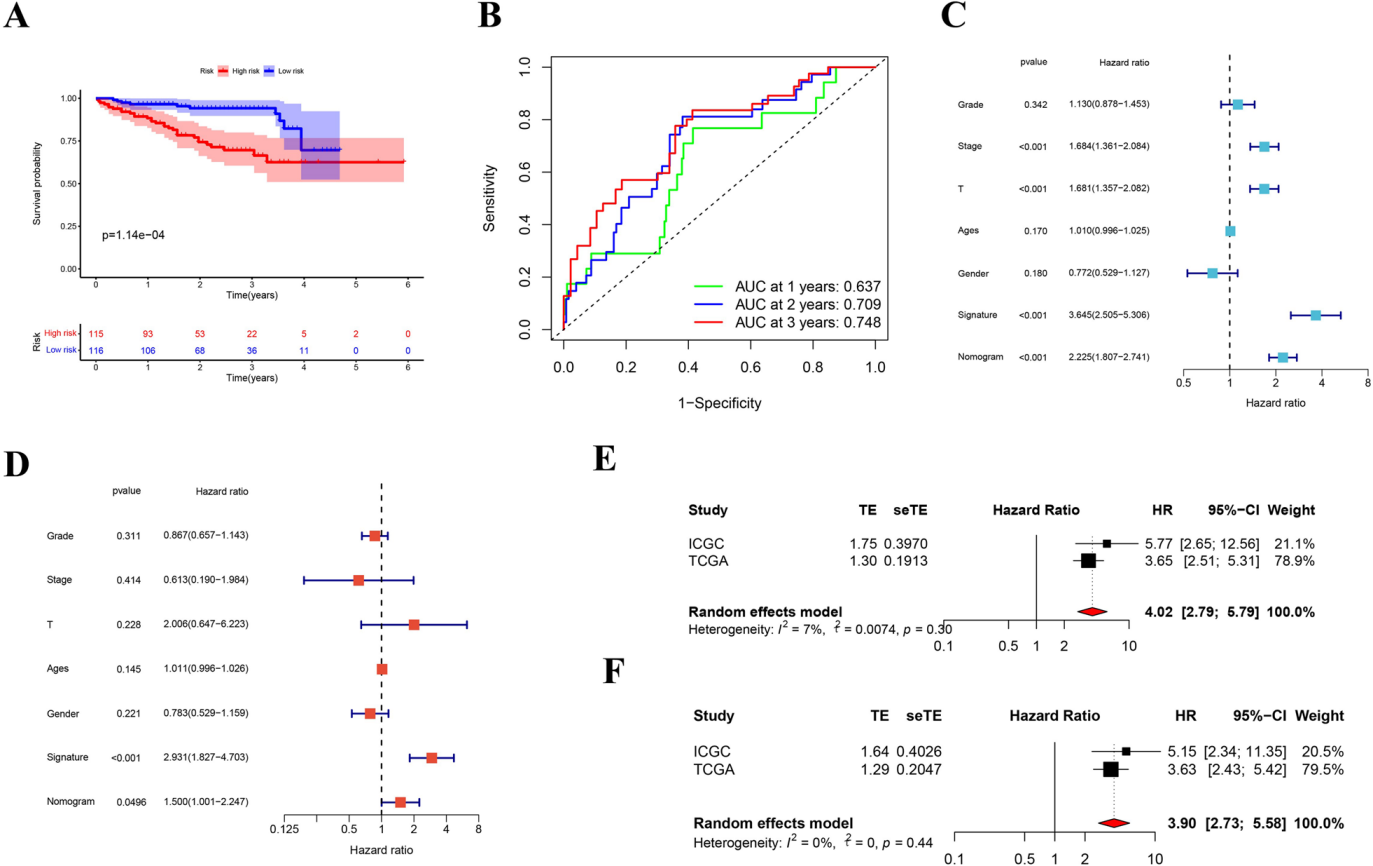
Prognostic value of the model. **A**, **B** Kaplan–Meier survival analysis and time-dependent ROC analysis to predict the overall survival of patients in the ICGC cohort. These predictions were based on the risk score. **C**, **D** Univariate and multivariate regression analyses of the ICGC cohort to examine the association between risk score and clinicopathological characteristics in terms of overall survival. Green represents univariate analysis; red represents multivariate analysis. **E**, **F** Forrest plots of TCGA and ICGC cohorts based on univariate and multivariate Cox analyses

Patients were categorized into either high- or low-risk groups based on a consistent cutoff value, depending on the risk score. Consistent with previous research, the high-risk group exhibited a significantly lower OS compared with the low-risk group (Fig. 5A). AUvalues for the risk score in predicting OS at 1, 2, and 3 years were 0.637, 0.709, and 0.748, respectively (Fig. 5B). Additionally, both univariate and multivariate Cox regression analyses demonstrated that the risk score could independently predict the prognosis (Fig. 5C, D).  More detailed information about univariate and multivariate Cox regression analyses were presented in Additional files 9, 10: supplementary table 3 and 4.

In addition, we conducted a meta-analysis of the ICGC and TCGA cohorts, which confirmed that the risk score was a reliable and independent predictor of OS in the patients with LIHC (Fig. 5E, F).

### Construction and validation of the integrated nomogram

**Fig. 6:**
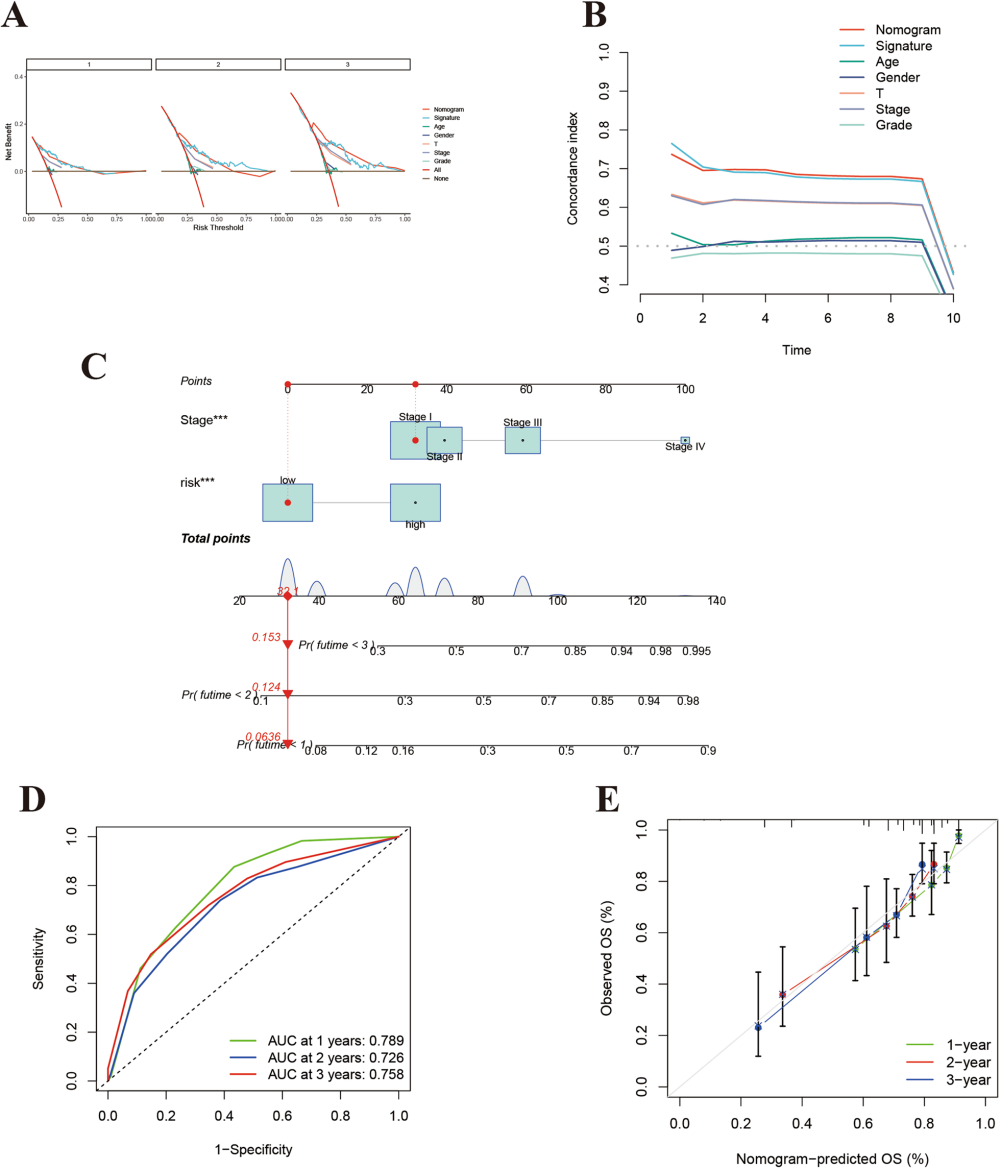
**A** Nomogram constructed on the basis of stage and risk score. **B** C-indices for risk score and clinicopathological characteristics. **C** Prognostic signature decision curve analysis for risk score and clinicopathological characteristics. **D** Time-dependent ROC curves for the sensitivity and specificity of the prognosis assessment for the whole cohort. **E** Calibration plots of overall survival (OS) at 1, 2, and 3 years

Next, a nomogram integrating the risk model and clinical features was constructed to more precisely predict the prognosis of patients. The constructed nomogram is presented in Fig. 6A; the risk scores and pathological characteristics were assigned specific scores based on their contribution to LIHC prognosis. Regarding the nomogram model diagnosis, the C-index (Fig. 6B) and decision curve analysis (Fig. 6C) indicated acceptable accuracy. The AUC for assessing the predictive values of the TCGA cohort nomogram for 1-, 2-, and 3-year OS were 0.789, 0.726, and 0.758, respectively (Fig. 6D). Moreover, calibration plots revealed consistent and stable alignment between the nomogram-predicted probabilities and actual observations for TCGA cohort 1-, 2-, and 3-year OS (Fig. 6E). Collectively, these results indicate that the constructed nomogram exhibited strong performance in predicting prognosis.

### Sensitivity to anticancer drugs between different risk groups

To guide clinical decision-making, we utilized risk scores to predict the sensitivity of high- and low-risk groups to common anticancer drugs, aiming to identify potential treatments for LIHC. Our findings indicated that the IC50 values of 10 drugs (Axitinib, AZD6244, AZD6482, BMS.536924, CGP.60474, Cyclopamine, Dasatinib, Docetaxel, Erlotinib, Pazopanib) were elevated in patients with higher risk scores. This suggests that lower risk scores are associated with increased sensitivity to these drugs, as illustrated in Additional file 2: Fig. S2A. Conversely, the IC50s of 10 drugs (CGP.082996, doxorubicin, epothilone B, gemcitabine, imatinib, and mitomycin. C, Paclitaxel, PHA.665752, S. Trityl. L. cysteine, and VX.680) were lower in high-risk patients (Additional file 2: Fig. S2B).

### Expression of six signature genes

**Fig. 7:**
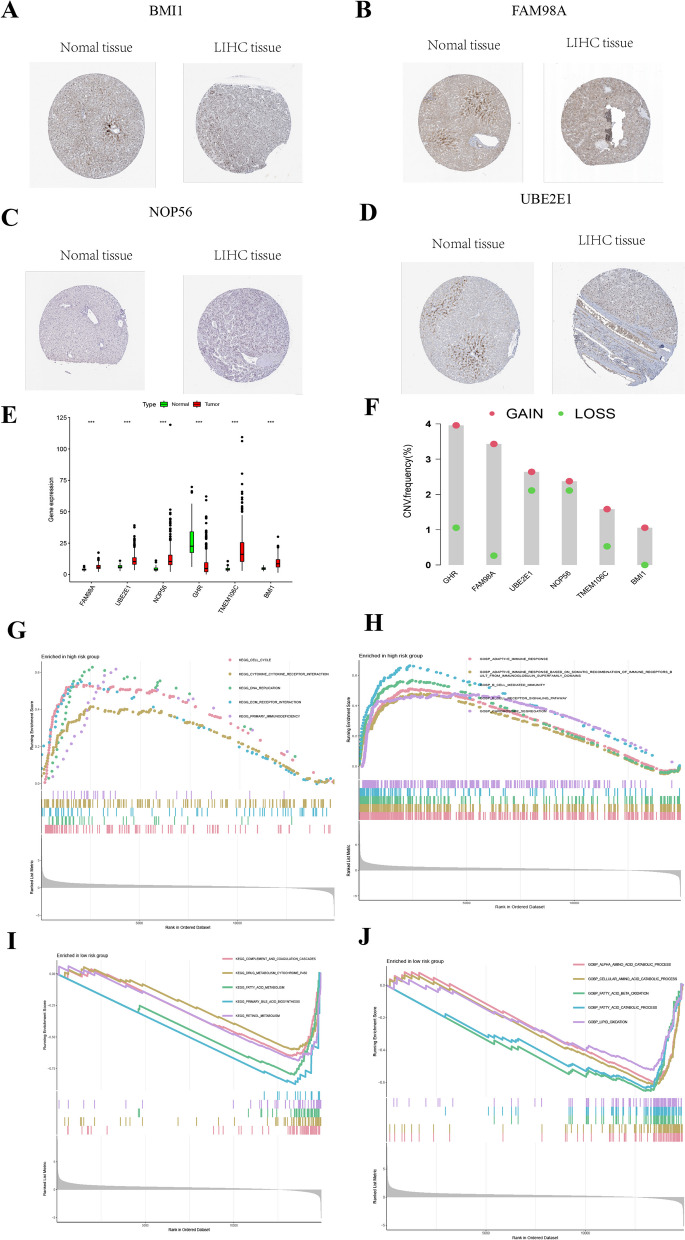
**A**–**D** Protein expression of BMI1, FAM98A, NOP56, and UBE2E1 in LIHC tissues and normal liver tissues. **E** Gene expression of six signature genes in LIHC tissues and normal liver tissues. **F** Copy number variation of six signature genes. **G**, **H** Enriched GO and KEGG pathways associated with DEGs of high risk-patients predicted by GSEA analysis. **I**, **J** Enriched GO and KEGG pathways associated with DEGs of low-risk patients predicted by GSEA analysis

The protein expression patterns of the six signature genes in LIHC and normal liver tissues were further explored using the HPA database. The results are shown in Fig. 7A (BMI1), Fig. 7B (FAM98A), Fig. 7C (NOP56), and Fig. 7D

(UBE2E1). IHC staining indicated that TMEM106C and GHR proteins were not expressed in either LIHC or normal tissues. BMI1, FAM98A, and NOP56 exhibited moderate expression in LIHC tissues, but low expression in normal liver tissues. Additionally, the protein UBE2E1 was not expressed in normal liver tissues but was moderately expressed in LIHC tissues.

### Expression and CNV frequency of the six signature genes

Next, we analyzed the expression patterns of the six signature genes using data from TCGA. There were significant differences in the expression levels of the six genes between tumor and normal tissues (*p* < 0.001; Fig. 7E), with five genes showing significant upregulation and one gene exhibiting significant downregulation. The frequency of copy number increase was higher than that of copy number loss (Fig. 7F).

### GSEA

KEGG pathway and GO enrichment analyses of the high- and low-risk groups (Fig. 7G–J) provided valuable insights into the potential molecular mechanisms underlying LIHC and offer promising avenues for future research.

### Correlation between immune cell infiltration, tumor stemness, and risk score

We explored the relationship between risk score and immune cell infiltration, revealing several noteworthy correlations. First, we found that the risk score exhibited a negative association with the stromal score, indicating that higher risk scores were associated with a reduced presence of intratumorally infiltrated stromal cells (Additional file 3: Fig. S3A). Moreover, the risk score was positively correlated with various immune cell types, including memory B cells, plasma B cells, M0 macrophages, M1 macrophages, M2 macrophages, myeloid dendritic cells, resting NK cells, T cell CD4 + memory T cells, follicular helper T cells, and regulatory T cells (Tregs). Conversely, there was a negative correlation with endothelial cells (Additional file 3: Fig. S3B). Furthermore, through Pearson’s correlation analysis, we identified a positive correlation between the risk score and RNA stemness score (RNAss; R = 0.3, *p* = 4.1e−09; Additional file 3: Fig. S3C), indicating that higher risk scores correspond to an increased presence of tumor stem cells.

### Benefits of ICI therapy in different risk groups

TIDE were used to analyze immune checkpoint inhibitor (ICI) therapy. High-risk patients exhibited lower TIDE scores, indicating that they might derive more significant benefits from ICI therapy (Additional file 3: Fig. S3D). Besides, supplementary analyses of ICI treatment response for different key genes were presented in Additional file 6: Figure S6.

### Expression of four DEGs in *Schistosoma japonicum* infected mice and LIHC cells

**Fig. 8:**
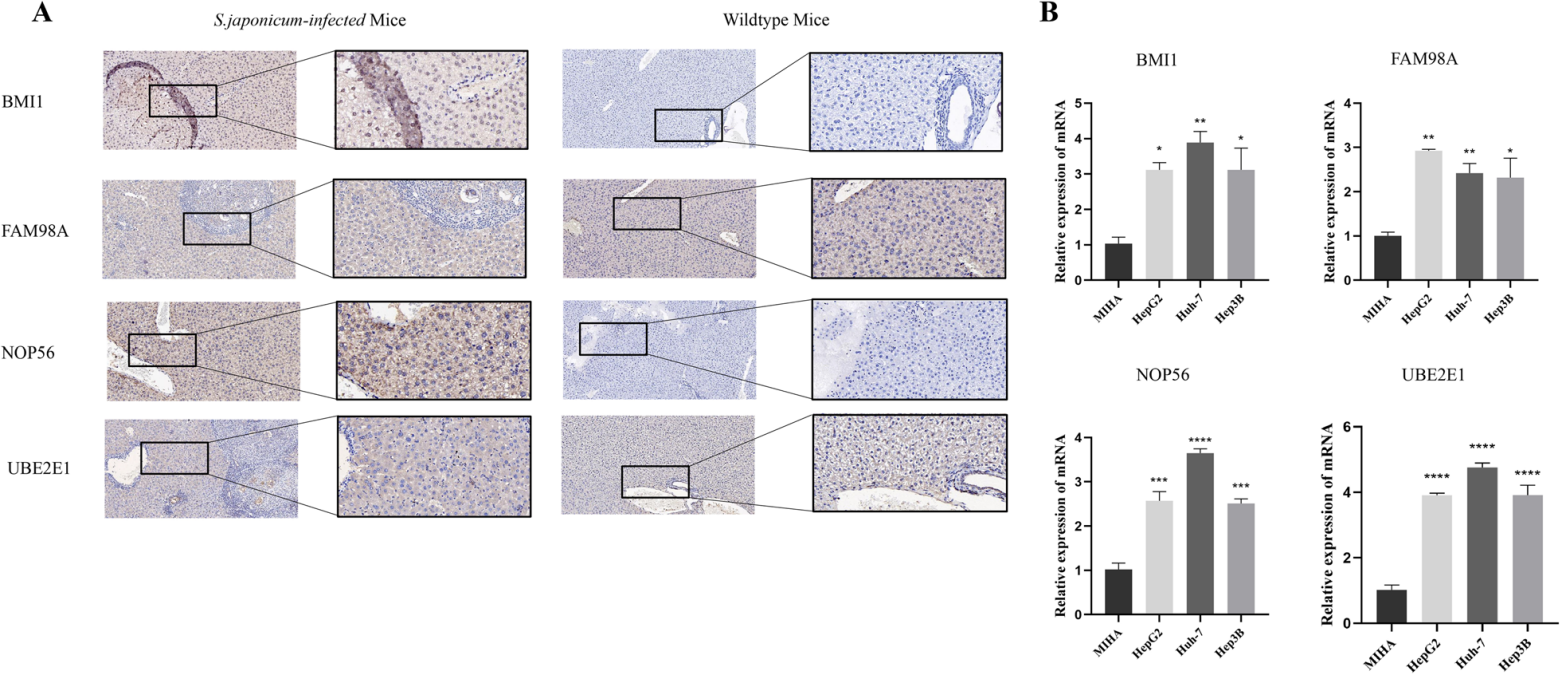
Expression of DEGS between normal tissues and LIHC tissues. **A** Expression levels of DEGs in schistosomiasis infection tissues, which are upregulated compared with wild-type tissues. **B** Expression levels of DEGs in LIHC cell lines, which are upregulated compared with the MIHA

Expression levels of BMI1, FAM98A, UBE2E1, and NOP56 were measured in *Schistosoma japonicum*-infected mice and LIHC cells. IHC results showed that the expression levels of BMI1, FAM98A, UBE2E1, and NOP56 were elevated in *Schistosoma japonicum*-infected mice compared with those in wild-type mice (Fig. 8A). The expression levels in LIHC cells (HepG2, Huh-7, and Hep3B cells) and normal hepatocytes (MIHA cells) detected by quantitative real-time polymerase chain reaction (PCR) were the same as those detected by IHC (Fig. 8B).

### BMI1 inhibits proliferation and induces apoptosis of LIHC Cells

Based on previous studies, BMI1 exhibits the most significant oncogenic effect among the four DEGs. In addition, we explored their biological functions. BMI1 was knocked down in HepG2 and Huh-7 cells by transfecting BMI1 siRNA into LIHC cells. The CCK-8 assay was used to investigate the effects of BMI1 on LIHC proliferation in BMI1 knockdown cells. As shown in Fig. 8A, B, BMI1 knockdown inhibited the growth of LIHC cells. Additionally, flow cytometry analysis revealed that LIHC cells with low BMI1 expression exhibited a larger G0/G1 population with arrest in S and G2/M phases (Additional file 4: Fig. S4A). Moreover, flow cytometry results indicated that BMI1 inhibited apoptosis in LIHC cells (Additional file 4: Fig. S4D).

To further investigate the potential role of BMI1 in regulating the invasion and migration abilities of LIHC cells, we conducted transwell (Additional file 5: Fig. S5A) and wound healing (Additional file 5: Fig. S5B) experiments after BMI1 knockdown. Invasion and migration were inhibited in BMI1 low expression cells.

In summary, our findings suggest that BMI1 plays a pivotal role in promoting the proliferation and cell cycle progression of LIHC cells, suppressing apoptosis and enhancing their invasion and migration capabilities.

## Discussion

The relationship between parasites and tumors has long been of research interest in the field of immune infections. Exploring the connection between schistosomiasis and liver hepatocellular carcinoma (LIHC) has posed challenges. Following Japan's announcement of complete schistosomiasis elimination, China is committed to eradicating the disease by 2030. The prevalence and intensity of *Schistosoma japonicum* infections in China are extremely low [18]. In 2019, approximately 30,170 people across China suffered from the disease, with only 5 new cases detected [50]. Although China has made significant progress in the prevention and control of schistosomiasis, *Schistosoma japonicum* remains a substantial threat to human health. [11, 47]. There is growing concern that the national control program may not achieve its ultimate goal, which is the complete elimination of the disease from China. It is also worth noting that schistosomiasis control efforts could be scaled back prematurely because of the mistaken belief that the disease is fully under control and no longer poses a significant risk to the public [45]. Even if China successfully eliminated schistosomiasis infection entirely, schistosomiasis patients reaching zero would not put an end to China's schistosomiasis control work. Therefore, it is insufficient to simply eliminate schistosomiasis. Moreover, the long-term impacts of schistosomiasis on the host need to be understood. Given the widespread history of schistosomiasis, the risk of overlooking the carcinogenic potential of schistosomiasis history is substantial. In this study, we investigated the roles of *Schistosoma japonicum* infection-associated genes in hepatocarcinogenesis.

Owing to the lack of a database that directly links *Schistosoma japonicum* infection to liver cancer, we proposed a novel approach. Liver cancer is the terminal stage of liver fibrosis, cirrhosis, and other chronic liver diseases. Therefore, we selected chronic liver disease as the bridge between schistosomiasis and liver cancer. First, we used the GEO database to identify DEGs between the livers of schistosomiasis-infected individuals and healthy livers. After removing the duplicates, 442 genes were identified. Then, 230 DEGs, which were clustered by the WGCNA algorithm using TCGA data and believed to be involved in LIHC development, were divided into turquoise and blue modules. The DEGs were assessed using random forest survival analysis and evaluated for prognostic importance. Among them, 17 DEGs with a gene scores of > 0.4 were considered representative. After eliminating 2 DEGs not shared between the ICGC and TCGA databases, 15 DEGs were divided into two subtypes by NMF analysis. We compared the differences in infiltrating immune cells, immune subtypes, tumor stromal scores, and survival between the two subtypes. The LASSO regression algorithm was used to select the 6 most representative DEGs from the 15 DEGs. Using TCGA and ICGC data as the training and test sets, respectively, we constructed a robust and feasible prediction model. Additionally, we established a nomogram based on staging characteristics and risk scores and employed calibration curves, receiver operating characteristic (ROC) curves, time-dependent C-index, and DCA to verify the accuracy of the model. Additionally, we considered drug sensitivity in patients with different risk scores for conventional chemotherapy and emerging immunotherapies. Owing to the availability of only four of the six genes in the HPA database, we further analyzed only four DEGs—BMI1, UBE2E1, FAM98A, and NOP56—in cancerous and normal tissues. Gene expression and copy number variations (CNV) can provide guidance for subsequent clinical immunotherapy. Furthermore, we performed GO and KEGG analyses of high- and low-risk groups to explore their enrichment pathways, aiding further investigation of potential molecular mechanisms. Finally, we used multiple algorithms to examine the extent of infiltration of various immune cells in different subgroups, and evaluated and analyzed the tumor microenvironment and stemness.

The transition from schistosome infection to liver cancer is a multistep process that involves various pathological and immune reactions and may progress through multiple clinical stages. The most common complication after infection is liver fibrosis, which has been extensively studied. However, the progression to advanced stages of the disease, whether it is a linear development from persistent liver fibrosis to cirrhosis and eventually liver cancer, or a network-like regulation where schistosome infection directly induces the occurrence of liver cancer, remains uncertain. Previously, the relationship was confirmed to be multidisciplinary and multilevel; however, no unified conclusion has been reached. This is because schistosomiasis infection and various liver diseases are long-term conditions that are accompanied by various interfering pathogenic factors, and current research methods suffer from specific errors. From the most general perspective, epidemiological evidence broadly demonstrates an increased incidence of liver cancer in patients with schistosomiasis infection [23, 26]. However, this does not exclude the presence of other pathogenic factors that often accompany schistosomiasis infection. Some scholars believe that the increased incidence of liver cancer caused by schistosomiasis infection is due to a combination of other factors, such as hepatitis B virus infection and alcohol abuse. However, Filgueira et al. pointed out that Mansoni schistosomiasis can independently promote liver cancer development even in the absence of other risk factors [15]. Thus, epidemiological studies that do not involve the molecular mechanisms cannot answer these questions. El-Tonsy et al. used various mouse models and compared intergroup tumor incidence and growth to demonstrate that schistosomiasis infection promotes the occurrence and development of liver cancer [13]. This greatly advanced research in the field, leaving only the exploration of the mechanisms, which requires a step-by-step progression, starting from observations at the tissue level, followed by the investigation of intricate molecular regulation within cells. At the tissue level, a study on the oncogenic mechanism of schistosomiasis found that schistosomiasis infection reduces the ability of the liver to detoxify carcinogens. Interestingly, some mutagens have a planar polycyclic aromatic structure that can tightly bind to heme-like deposits formed in the infected liver. These findings suggest that certain carcinogens may be retained longer in infected animals than in uninfected animals, thereby increasing the exposure of animals to carcinogens, leading to the occurrence of liver cancer [7, 22]. With the rapid development of molecular biology, in-depth research on the oncogenic mechanisms of schistosomiasis is becoming increasingly important. Roderfeld et al. showed that the substances released by liver tissue-captured schistosome eggs permanently activated oncogenes related to liver cell carcinoma, such as c-Jun and related transcription factors (STAT3) [42]. However, these studies began with biomarkers and failed to explain the oncogenicity of schistosomiasis through a complete pathway. Therefore, the specific upstream molecular changes caused by schistosomiasis need to be elucidated.

In tracing the causes of liver cancer, it is difficult to confirm changes in specific molecular targets caused by schistosomiasis infection only through changes in some recognized oncogenes, making individualized diagnosis and treatment difficult to achieve. This study builds on the pioneering results of previous studies, focusing on further exploring multiple targets (suspected molecules) at a deeper level. We propose that before the recognized oncogenic pathways are activated, four key DEGs (BMI1, NOP56, UBE2E1, and FAM98A) are aberrantly expressed, revealing the different prognoses of patients with liver cancer. NOP56, UME2E1, and FAM98A play important roles in the life cycle of cells and participate in regulating key processes such as proliferation, division, and differentiation. Their functions may involve the regulation of cell cycle control pathways, which are crucial for understanding the mechanisms of cell cycle regulation and occurrence of related diseases [6, 39, 41]. NOP56 and UBE2E1 have been identified as molecular markers for endometrial cancer (Bradfield et al. 2020). We believe that these three genes were selected because they are involved in inhibiting apoptosis and promoting the proliferation of liver cancer cells during further development of hepatocellular carcinoma. However, we considered the most crucial initiating stage to be mediated by BMI1, as it is a confirmed oncogene. Further experiments were designed to investigate the relevance of these results.

B-lymphoma Mo-MLV insertion region 1 (BMI1) encodes a crucial Polycomb group (PcG) protein that is involved in histone modification. This is achieved by forming a stable heterodimer with RING1B and subsequently ubiquitylating histone H2A. BMI1 has been recognized as a proto-oncogene [43] that encodes a protein consisting of 324 amino acids, is primarily localized in the nucleus, and has been implicated in the development of mouse pre-B-cell lymphomas [44]. Accumulating evidence suggests that BMI1 is closely associated with the onset, progression, and outcome of various human malignancies [17, 25, 30]. Previous reports have indicated that BMI-1 is overexpressed in gastric, ovarian, breast, head and neck, pancreatic, and radiation-induced lung cancers, as well as in primary hepatocellular carcinoma (HCC) and endometrial carcinoma [8, 14, 17, 21, 28, 36, 48, 49]. Furthermore, overexpression of BMI-1 has also been found in patients with myelodysplastic syndromes, chronic myelogenous leukemia, acute myeloid leukemia, and lymphoma [1, 35, 37, 38]. BMI1 is overexpressed in one-third of patients with LIHC and is considered a key target for LIHC treatment [29]. Small molecule inhibitors targeting BMI1 have shown great potential in the treatment of other tumors. Kreso and his colleagues found that PTC-209 could effectively inhibit the self-renewal ability of colorectal cancer initiating cells (CIC) by intratumoral administration, leading to a significant reduction in CICs and impaired tumor growth [27]. PTC-028 is a second-generation BMI1 inhibitor with optimized pharmacological properties. Compared with PTC-209, PTC-028 differs in regulatory mechanisms, BMI1 turnover rate, cellular ATP reduction, mitochondrial ROS production, as well as administration route and dosage [12, 24, 46]. In vivo and in vitro experimental results show that PTC-028 can significantly inhibit the self-renewal ability of tumor cells and significantly reduce local and metastatic tumor burden [4, 5]. PTC-596 is another commonly used small molecule BMI1 inhibitor that can selectively induce massive death of acute myeloid leukemia (AML) stem/progenitor cells without affecting normal hematopoietic cells [40]. Therefore, confirming that BMI1 is a crucial factor involved in schistosomiasis-induced liver cancer is very important. Targeted small molecule drugs may reverse carcinogenesis. In this study, we considered that BMI1 may be key to unlocking the role of Schistosoma infection in the development of liver cancer. In addition, we discussed prognosis, immune escape, drug sensitivity, and other factors through bioinformatics analysis, which can guide clinical immunotherapy. Finally, we explored the expression changes and biological functions of BMI1 during schistosome infection and LIHC development by molecular biological methods.

This study has some limitations. First, owing to a lack of sequencing results for schistosomiasis-induced liver cancer, we utilized datasets of a series of liver diseases induced by schistosomiasis for indirect research, selecting genes positively correlated with liver cancer. Cases of liver cancer caused solely by schistosomiasis are difficult to obtain; however, in the future, genomic detection in animal models may provide another source of data. Alternatively, detecting the four genes selected in our study in individuals with schistosomiasis may be highly meaningful. Second, the evidence supporting our conclusions, especially from the molecular biology persecutive, is insufficient. Further detailed molecular mechanisms linking schistosomiasis infection and the development of LIHC need to be explored. In the future, we will continue to conduct in-depth research on this topic.

## Conclusions

We conducted the first comprehensive investigation of the prognostic signature in *Schistosoma japonicum* infection-associated LIHC patients and the underlying molecular mechanisms. The prognostic signature identified herein not only enhances our understanding of the complex interplay between parasitic infection, host immune response, and LIHC development, but also has the potential to guide clinical decision-making and personalized treatment strategies for LIHC patients with a history of schistosomiasis. Future research should focus on validating these findings in larger, independent cohorts and investigating the underlying biological processes, which may pave the way for novel therapeutic targets and strategies for the treatment of LIHC patients with a history of schistosomiasis infection.

### Supplementary Information

**Additional file 1**. **Fig. S1**. Genes common to both TCGA and ICGC datasets in the non-negative matrix factorization for identifying disease subtypes. **A** Distribution of cophenetic, RSS, dispersion, etc., with a rank of 2–10. **B** Consensus map of NMF clustering. **C** Consensus maps of NMF clustering.

**Additional file 2**. **Fig. S2**. Differences in chemotherapy sensitivity between low- and high-risk patients.

**Additional file 3**. **Fig. S3**. **A** Spearman correlation analysis of the stromal scores. **B** Correlation analysis of immune infiltration using multiple softwares. **C** Spearman correlation analysis of RNAss scores. **D** TIDE scores of high- and low-risk patients.

**Additional file 4**. **Fig. S4**. BMI1 knockdown decreases the proliferation rate of LIHC cells. **A**, **B** CCK8 assay results showing that BMI1 knockdown decreases the proliferation rate of HepG2 and Huh-7 cells. The data are expressed as the mean ± SD. *p < 0.05, **p < 0.01, ***p < 0.001 (t test). **C** Flow cytAometry results for LIHC cells transfected with BMI1-siRNA showing a larger G0/G1 population and arrested S and G2/M phases. **D** Flow cytometry confirming the ability of BMI1 to inhibit cells apoptosis.

**Additional file 5**. **Fig. S5**. BMI1 knockdown inhibits the invasion ability of LIHC cells. **A** The invasion ability of LIHC cells is inhibited by BMI1 knockdown. The data are presented as the mean ± SD. **p < 0.01, ***p < 0.001, ****p < 0.0001 (t test). (B) Images representing cells at 0 and 48 h post-transfections; BMI1 knockdown hampers the migration of LIHC cells.

**Additional file 6**. **Fig. S6**. ICB (Immune Checkpoint Blockade) response in various cancers. **A** ROC analysis of Signature, BST2, TIDE (Tumor Immune Dysfunction and Exclusion), MSI. Score, TMB (Tumor Mutational Burden), CD274, CD8, IFNG (Interferon Gamma), T. Clonality, B. Clonality and Merck18 in predicting the ICB response in various cancers. **B** AUC analysis of different key genes undergoing various ICI therapy were presented as oncology swimlane diagrams.

**Additional file 7**. **Table S1**. Basic clinicopathological information of TCGA.

**Additional file 8**. **Table S2**. Basic clinicopathological information of ICGC.

**Additional file 9**. **Table S3**. The detailed information of univariate Cox regression analysis.

**Additional file 10**. **Table S4**. The detailed information of multivariate Cox regression analysis

## Data Availability

Data included in article/supp. material/referenced in article.
