# Supplementary figures and images for: A prognostic model for Schistosoma japonicum infection-associated liver hepatocellular carcinoma: strengthening the connection through initial biological experiments
Source: Infect Agent Cancer. 2024 Mar 21;19:10. doi: 10.1186/s13027-024-00569-4 (PMC10956344; doi:10.1186/s13027-024-00569-4)

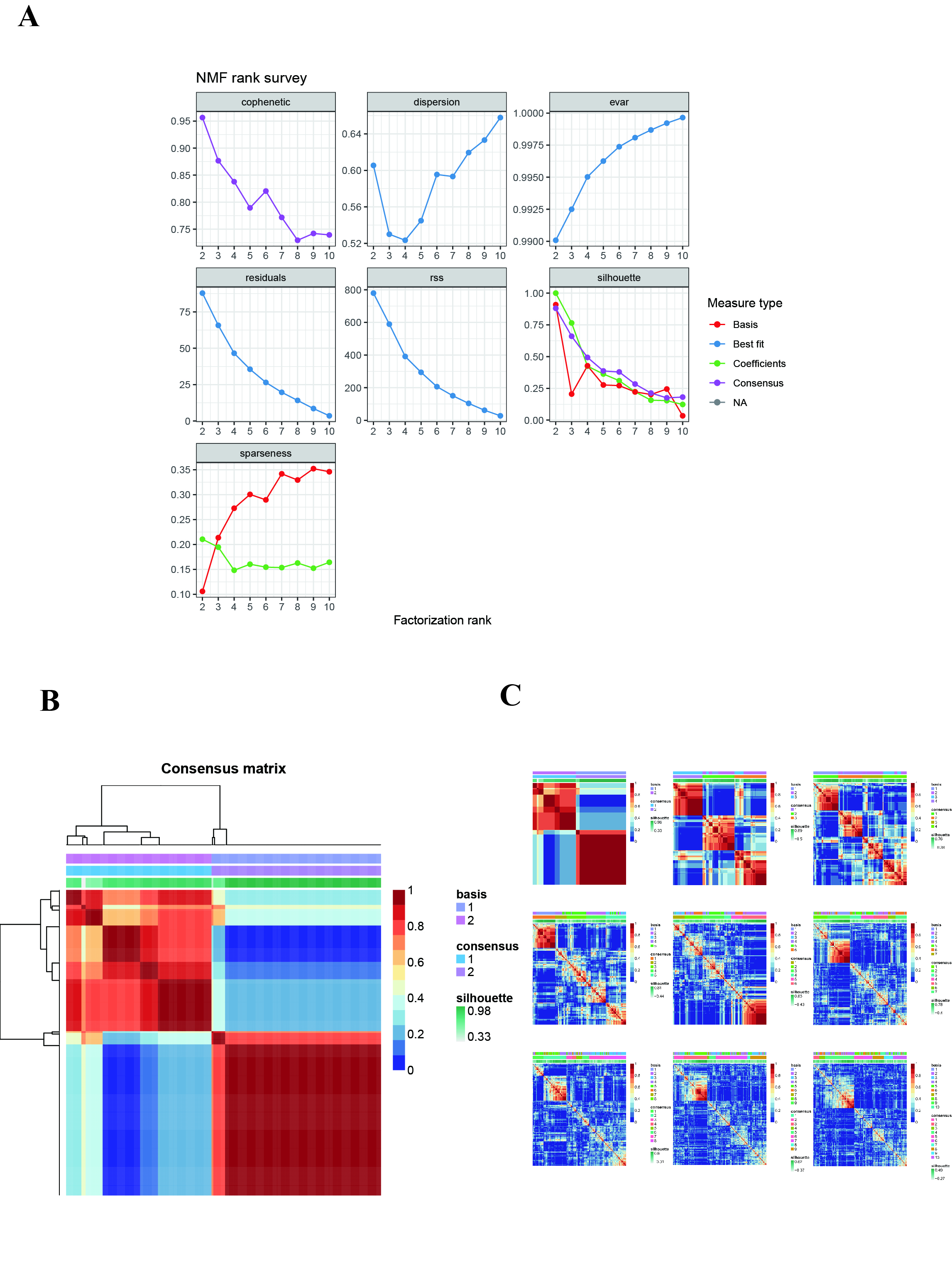

Supplement: Supplementary file 1 — Additional file 1. Fig. S1. Genes common to both TCGA and ICGC datasets in the non-negative matrix factorization for identifying disease subtypes. A Distribution of cophenetic, RSS, dispersion, etc., with a rank of 2–10. B Consensus map of NMF clustering. C Consensus maps of NMF clustering. [file 13027_2024_569_MOESM1_ESM.jpg]

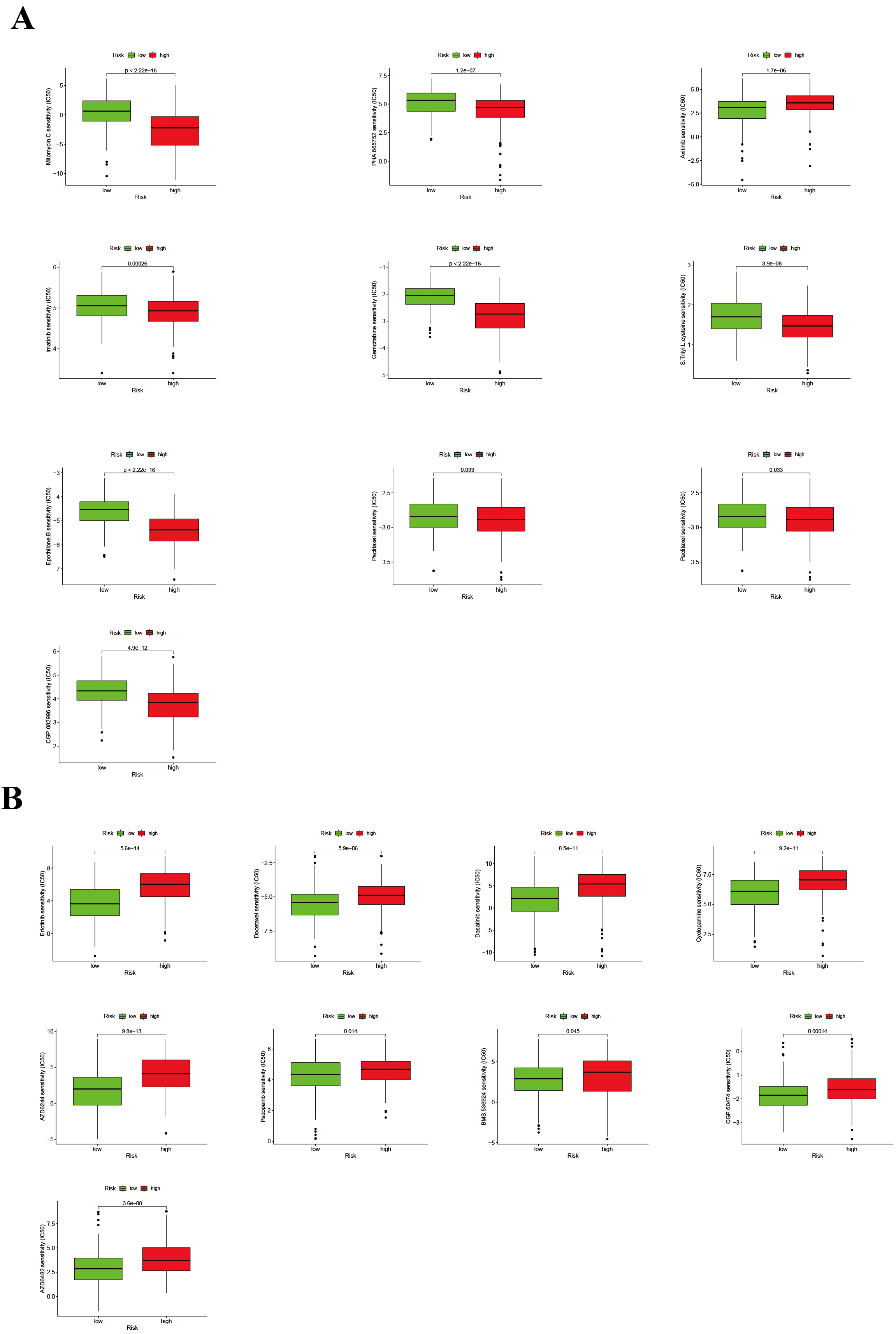

Supplement: Supplementary file 2 — Additional file 2. Fig. S2. Differences in chemotherapy sensitivity between low- and high-risk patients. [file 13027_2024_569_MOESM2_ESM.jpg]

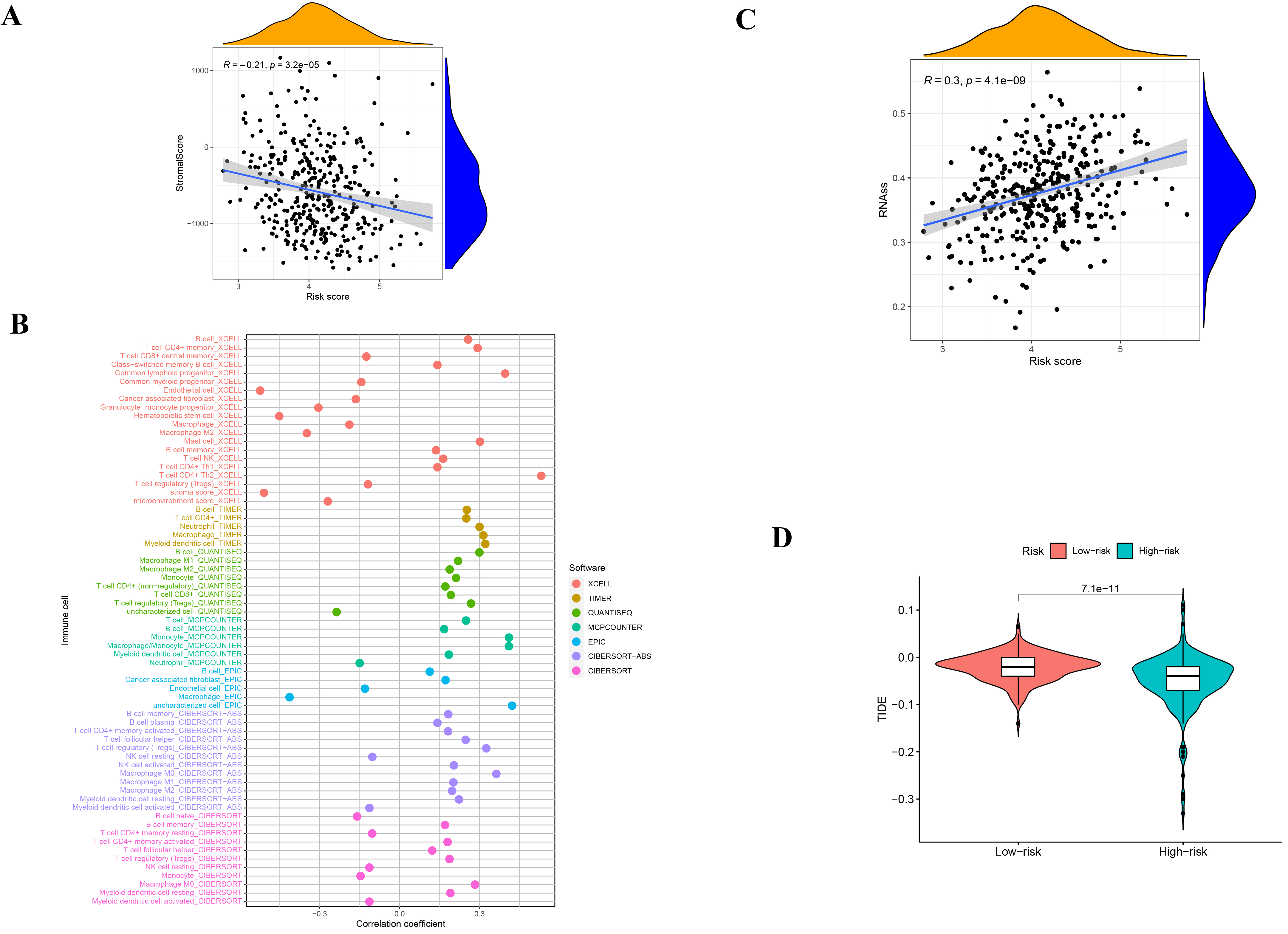

Supplement: Supplementary file 3 — Additional file 3. Fig. S3. A Spearman correlation analysis of the stromal scores. B Correlation analysis of immune infiltration using multiple softwares. C Spearman correlation analysis of RNAss scores. D TIDE scores of high- and low-risk patients. [file 13027_2024_569_MOESM3_ESM.jpg]

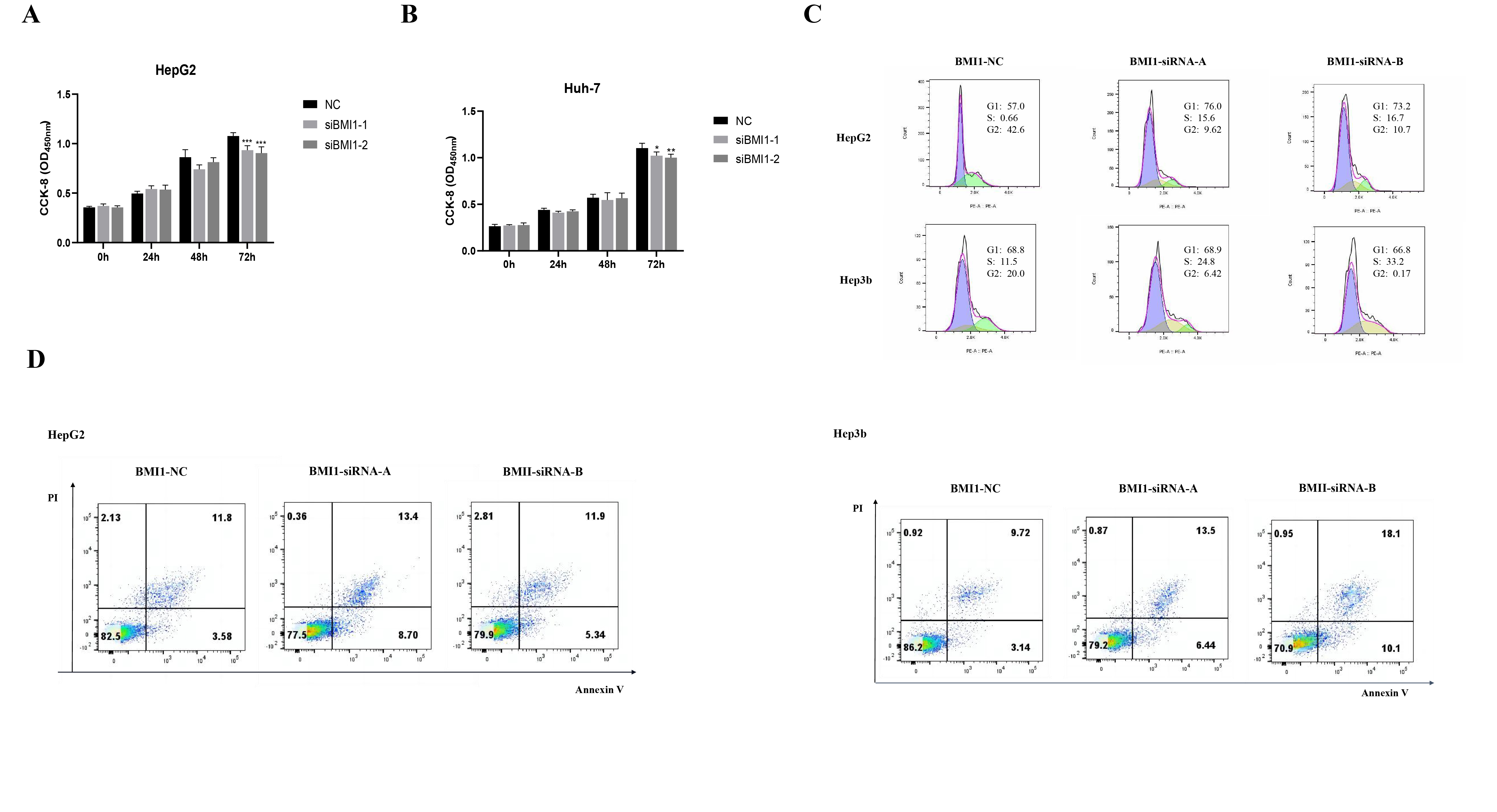

Supplement: Supplementary file 4 — Additional file 4. Fig. S4. BMI1 knockdown decreases the proliferation rate of LIHC cells. A, B CCK8 assay results showing that BMI1 knockdown decreases the proliferation rate of HepG2 and Huh-7 cells. The data are expressed as the mean ± SD. *p < 0.05, **p < 0.01, ***p < 0.001 (t test). C Flow cytAometry results for LIHC cells transfected with BMI1-siRNA showing a larger G0/G1 population and arrested S and G2/M phases. D Flow cytometry confirming the ability of BMI1 to inhibit cells apoptosis. [file 13027_2024_569_MOESM4_ESM.jpg]

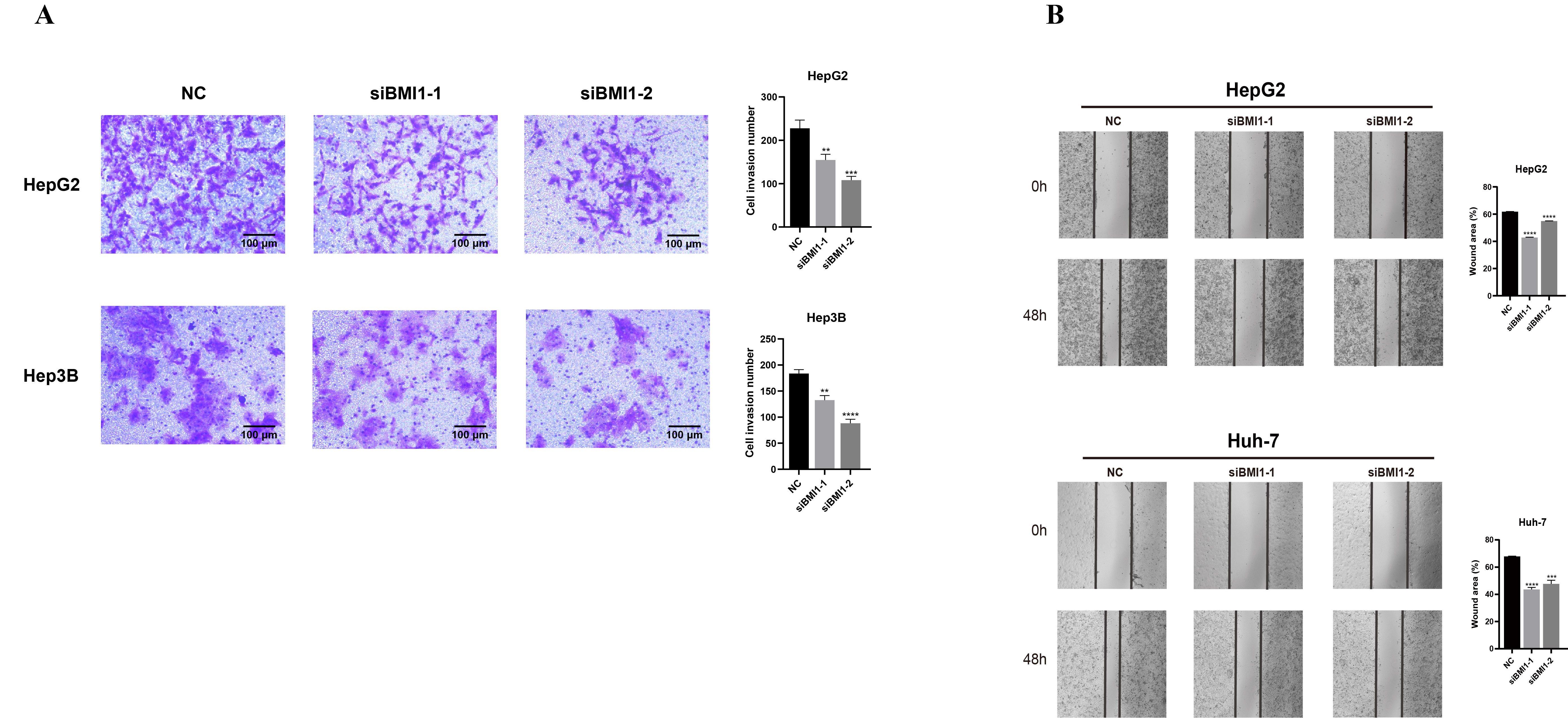

Supplement: Supplementary file 5 — Additional file 5. Fig. S5. BMI1 knockdown inhibits the invasion ability of LIHC cells. A The invasion ability of LIHC cells is inhibited by BMI1 knockdown. The data are presented as the mean ± SD. **p < 0.01, ***p < 0.001, ****p < 0.0001 (t test). (B) Images representing cells at 0 and 48 h post-transfections; BMI1 knockdown hampers the migration of LIHC cells. [file 13027_2024_569_MOESM5_ESM.jpg]

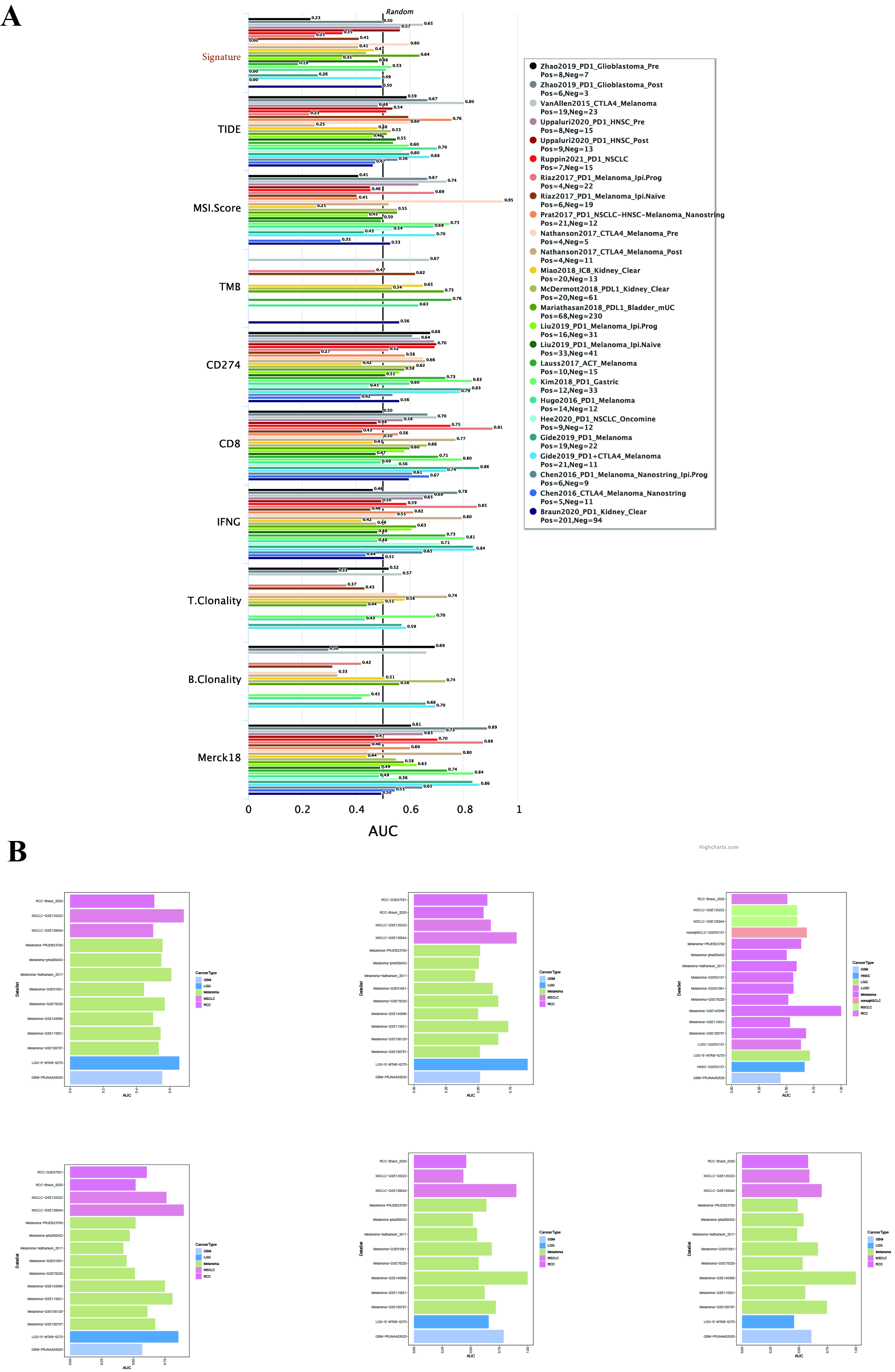

Supplement: Supplementary file 6 — Additional file 6. Fig. S6. ICB (Immune Checkpoint Blockade) response in various cancers. A ROC analysis of Signature, BST2, TIDE (Tumor Immune Dysfunction and Exclusion), MSI. Score, TMB (Tumor Mutational Burden), CD274, CD8, IFNG (Interferon Gamma), T. Clonality, B. Clonality and Merck18 in predicting the ICB response in various cancers. B AUC analysis of different key genes undergoing various ICI therapy were presented as oncology swimlane diagrams. [file 13027_2024_569_MOESM6_ESM.jpg]
