# Supplementary material for: A prognostic model for Schistosoma japonicum infection-associated liver hepatocellular carcinoma: strengthening the connection through initial biological experiments
Source: Infect Agent Cancer. 2024 Mar 21;19:10. doi: 10.1186/s13027-024-00569-4 (PMC10956344; doi:10.1186/s13027-024-00569-4)
Supplement: Supplementary file 9 — Additional file 9. Table S3. The detailed information of univariate Cox regression analysis. [file 13027_2024_569_MOESM9_ESM.docx]

**Supplementary Table3** The detailed information of univariate Cox regression analysis

| id | HR | HR.95L | HR.95H | P value |
| --- | --- | --- | --- | --- |
| Grade | 1.12966015175578 | 0.878300750042528 | 1.45295567424155 | 0.342407655203698 |
| Stage | 1.68424831687491 | 1.36092766923214 | 2.0843814532013 | <0.0001 |
| T | 1.68090155178178 | 1.35735595130238 | 2.08156896801565 | <0.0001 |
| Ages | 1.01020283572763 | 0.995663696982776 | 1.02495428165621 | 0.16993121287137 |
| Gender | 0.77215383301147 | 0.529001049774888 | 1.12707062129276 | 0.180235316432917 |
| Signature | 3.64531095101717 | 2.50452460608366 | 5.30571426502559 | <0.0001 |
| Nomogram | 2.22543569990293 | 1.806740168277 | 2.74116009670912 | <0.0001 |
