# Supplementary material for: A prognostic model for Schistosoma japonicum infection-associated liver hepatocellular carcinoma: strengthening the connection through initial biological experiments
Source: Infect Agent Cancer. 2024 Mar 21;19:10. doi: 10.1186/s13027-024-00569-4 (PMC10956344; doi:10.1186/s13027-024-00569-4)
Supplement: Supplementary file 10 — Additional file 10. Table S4. The detailed information of multivariate Cox regression analysis [file 13027_2024_569_MOESM10_ESM.docx]

**Supplementary Table4** The detailed information of multivariate Cox regression analysis

| id | HR | HR.95L | HR.95H | P value |
| --- | --- | --- | --- | --- |
| Grade | 0.866861242218735 | 0.657464492700608 | 1.14294904379452 | 0.31114653024226 |
| Stage | 0.613315267517241 | 0.189548060891558 | 1.98448676077433 | 0.414498814913574 |
| T | 2.00599764296215 | 0.646592466717982 | 6.22343554974456 | 0.22815737632283 |
| Ages | 1.01096820621041 | 0.996263165902995 | 1.02589029580545 | 0.14451799536055 |
| Gender | 0.78280837024708 | 0.528878317279019 | 1.15865771862529 | 0.220986262642395 |
| Signature | 2.93143016560139 | 1.8271283959081 | 4.70316308095404 | <0.0001 |
| Nomogram | 1.49963621877929 | 1.0006617060393 | 2.24742165619189 | 0.0496265784444772 |
